# A Survey of the FDA's AERS Database Regarding Muscle and Tendon Adverse Events Linked to the Statin Drug Class

**DOI:** 10.1371/journal.pone.0042866

**Published:** 2012-08-22

**Authors:** Keith B. Hoffman, Christina Kraus, Mo Dimbil, Beatrice A. Golomb

**Affiliations:** 1 AdverseEvents, Inc., Healdsburg, California, United States of America; 2 Department of Medicine, University of California San Diego, La Jolla, California, United States of America; 3 Department of Family and Preventive Medicine, University of California San Diego La Jolla, California, United States of America; University of British Columbia, Canada

## Abstract

**Background:**

Cholesterol management drugs known as statins are widely used and often well tolerated; however, a variety of muscle-related side effects can arise. These adverse events (**AEs**) can have serious impact, and form a significant barrier to therapy adherence. Surveillance of post-marketing AEs is of vital importance to understand real-world AEs and reporting differences between individual statin drugs. We conducted a review of post-approval muscle and tendon AE reports in association with statin use, to assess differences within the drug class.

**Methods:**

We analyzed all case reports from the FDA AE Reporting System (**AERS**) database linking muscle-related AEs to statin use (07/01/2005–03/31/2011). Drugs examined were: atorvastatin, simvastatin, lovastatin, pravastatin, rosuvastatin, and fluvastatin.

**Results:**

Relative risk rates for rosuvastatin were consistently higher than other statins. Atorvastatin and simvastatin showed intermediate risks, while pravastatin and lovastatin appeared to have the lowest risk rates. Relative risk of muscle-related AEs, therefore, approximately tracked with per milligram LDL-lowering potency, with fluvastatin an apparent exception. Incorporating all muscle categories, rates for atorvastatin, simvastatin, pravastatin, and lovastatin were, respectively, 55%, 26%, 17%, and 7.5% as high, as rosuvastatin, approximately tracking per milligram potency (Rosuvastatin>Atorvastatin>Simvastatin>Pravastatin≈Lovastatin) and comporting with findings of other studies. Relative potency, therefore, appears to be a fundamental predictor of muscle-related AE risk, with fluvastatin, the least potent statin, an apparent exception (risk 74% vs rosuvastatin).

**Interpretation:**

AE reporting rates differed strikingly for drugs within the statin class, with relative reporting aligning substantially with potency. The data presented in this report offer important reference points for the selection of statins for cholesterol management in general and, especially, for the rechallenge of patients who have experienced muscle-related AEs (for whom agents of lower expected potency should be preferred).

## Introduction

We sought to analyze the FDA Adverse Event Reporting System (**AERS**) database in order to: (i) examine relative rates of statin side effects across muscle and tendon categories, and (ii) determine if meaningful safety differences might exist *between* the six main statin drugs.

While others have analyzed controlled clinical trials undertaken with this class of drugs, this analysis was designed to assess links between these drugs and adverse events in large, heterogeneous “real-world” patient populations, by analyzing over seven-years of FDA adverse event case reports.

Many studies have focused on one serious statin side effect, rhabdomyolysis. We examined rhabdomyolysis, but also included less devastating muscle-related side effects. These are important in their own right, due to their greater frequency, significant effects on quality of life, and impact on statin therapy non-compliance.

Our study employed both automated and manual data analysis methods in order to obtain all relevant case reports within the FDA AERS database from July 1, 2005 to March 31, 2011.

Statins are among the most widely taken prescription medications in the world. They are intended to reduce the risk of cardiovascular disease, the leading cause of death in most industrialized nations. A shared mode of action is the inhibition of 3-hydroxy-3-methyl glutaryl coenzyme A reductase (HMG-CoA reductase), a key enzyme in the cholesterol biosynthetic pathway. Statins have a strong efficacy record in reducing cholesterol, cardiovascular events, and (in secondary prevention for men under age 70), deaths [1], [2], [3].

However, dose-dependent side effects occur across the statin class [4], [5], [6]. Appreciable occurrences of muscle-related side effects were not uncovered during prerelease clinical testing of statins. In part this may be because factors that benefit cost, efficiency, and human research subject protections in clinical trials can impede the identification of adverse effects. Such factors include: (i) relative homogeneity of research subjects, (ii) self-selection of more robust subjects, and (iii) relative exclusion of elderly, subjects with comorbidities, and individuals with potential drug-drug interactions. Accordingly, side effects, including a range of muscle and tendon disorders extending from myalgia to life-threatening rhabdomyolysis, became evident primarily after the drugs won FDA approval. (For a review of suspected adverse events across the statin drug class, risk factors, and potential drug interactions that raise risk of statin myopathy, see Golomb and Evans, 2008 [7].) Exemplifying this, the elevated occurrence of rhabdomyolysis with cerivastatin (Baycol) [8] culminated in numerous deaths and the withdrawal of cerivastatin from the market. More recently, the FDA announced new safety recommendations for high dose simvastatin, citing an “increased risk of myopathy when using the 80 mg dose of simvastatin.” This warning was issued only after many years of clinical use of simvastatin, indeed among the best-selling prescription drugs, and five years after its loss of patent protection. (http://www.fda.gov/NewsEvents/Newsroom/PressAnnouncements/ucm258338.htm).

When a drug safety problem is important enough to merit regulatory action, earlier detection is presumably better, enabling more adverse events to be forestalled. Careful post-approval monitoring for adverse events is therefore vital to the continuing drug evaluation process. Systematic tools may facilitate the analysis of the vast archive that is the FDA AERS database. Accordingly, we performed a comprehensive analysis of AERS, and accompanying case report forms, to identify potentially important statin-related adverse events by combining manual searching techniques with a new AERS searching tool (the “RxFilter™” developed by AdverseEvents, Inc). While the use of the RxFilter™ significantly speeds and helps organize AERS searches, all the individual case reports that were analysed here can be obtained without the use of the searching tool. Emphasis was placed on muscle and tendon disorders, including: myopathy (general), myalgia, myositis, and rhabdomyolysis.

## Methods

Data were obtained from the FDA AERS database from case reports received by the FDA between July 1, 2005 and March 31, 2011. Drugs selected for analysis were: atorvastatin (Lipitor), simvastatin (Zocor), lovastatin (Mevacor), pravastatin (Pravachol), rosuvastatin (Crestor), fluvastatin (Lescol), and generic equivalents and foreign designations. We performed a detailed search of the AERS database, and accompanying case report forms, in order to identify potentially important treatment-related side effects. Steps include the following:

### I. Reorganizing Drug Name(s)

The AERS database was reorganized in order to accurately identify and aggregate all case reports for each marketed drug. Each drug name variant (including generic names, names outside the United States, misspellings, dosage descriptions, etc., as originally entered in the AERS database) was consolidated into one common name. For example, in the AERS database, atorvastatin has 810 separate designations, all of which are combined into a single name. The analysis herein included all such variants for each of the six drugs (see **File S1** for full name listings).

### II. Finding Adverse Event Case Reports

To determine the number of case reports associated with each drug, we cross-referenced the consolidated name as both the “primary” and “all” suspect in the FDA AERS database. We analyzed single adverse events as well as multiple events grouped into custom search lists. Such side effect categories are listed, along with the exact adverse event search terms used.

### III. Estimated Prescription Rates

To estimate relative risks across the statins, we normalized the precribing frequency for each drug by summing quarterly new prescription totals (“NRx”) provided by IMS Health during the applicable time period. Over the time period from July 1, 2005 to March 31, 2011 summed NRx totals were: simvastatin 122,377,000; atorvastatin 105,289,000; rosuvastatin 35,505,000; lovastatin 26,345,000; pravastatin 27,843,000; and fluvastatin 3,238,000 [9], [10], [11], [12]. Relative prescribing ratios were, accordingly: simvastatin, 1.00; atorvastatin, .8604; rosuvastatin, .2901; lovastatin, .2153; pravastatin, .2275; and fluvastatin, .0265. We also conducted a sensitivity analysis, employing the peak annual prescription figure for each drug between 2004 and 2010 (data not shown).

Duplicate case report forms (for example, those that describe the same adverse event from the same patient) were omitted from analysis. Displayed data represent the number of case report forms that link the specific adverse events with the noted statin drug as both “primary” and “all” suspect (as defined in the FDA AERS database).

A “ranked risk” calculation was derived by dividing the number of “primary suspect” and “all suspect” adverse events for each statin by its prescribing ratio during the time period of July 2005 through March 2011. In each category, the statin with the highest risk rate was designated as having a “ranked risk” value of 100. The remaining five drugs are comparison-ranked to that drug.

The purposefully broad inclusion parameters of the “Joints and Tendons,” “Muscle Atrophy and Injury,” and “Muscle Coordination and Weakness” adverse event categories represent an attempt to capture as many relevant potential cases of statin-related muscle/tendon side effects as possible.

Rosuvastatin was approved for US marketing in 2003, and is therefore the newest of the statins studied here; it is possible that reporting rates might be higher following market launch and diminish with time. To assess whether adverse event reporting rates were disproportionately high newly following the introduction of rosuvastatin, we collected yearly “primary suspect” case report totals linking all the statin drugs to: (i) rhabdomyolysis, (ii) a combined category of myalgia, myopathy, and myositis, and (iii) a non-muscle-related side effect, nausea.

#### Outcome Measures

We also collected “outcome measures,” such as death, disability, hospitalization, etc. (as defined within the FDA AERS database) for the main adverse event categories listed in this study.

#### Case Reports Analysis - Reporter Identification

In order to determine the relative impact of various reporting sources we cross-referenced reporter identification categories (as inputted into AERS) with the major adverse event categories queried in this study.

## Results

Cases: there were a total of 39,007 “primary” and 147,789 “all” suspect case reports listed within the AERS database, across all adverse event types. Individual case report totals for muscle and tendon-related adverse events are shown in **Table 1**. Rosuvastatin and fluvastatin appeared to be consistently linked to higher adverse event relative risks than other commonly used statins, while atorvastatin and simvastatin showed intermediate risks, and pravastatin and lovastatin appeared to have the lowest risk rates. Relative risks, therefore, approximately tracked with per milligram (and as-prescribed) potency [13], though there were some apparent departures. Summing primary adverse effects across muscle categories, normed to prescribing rates, rosuvastatin had the highest ranked risk incorporating all muscle categories. Designating rosuvastatin's relative risk for combined categories as 100%, comparative rates for atorvastatin, simvastatin, pravastatin, and lovastatin were, respectively, 55%, 26%, 17%, and 7.5%. Thus, rates approximately track per mg potency and maximum prescribed potency (expected to relate to as-prescribed potency), comporting with findings of other studies that have used different approaches [5]. Thus, relative potency appears to be a fundamental predictor of adverse effect reporting risk. Fluvastatin was a notable exception. This agent, bearing the lowest per-milligram potency, was relatively rarely prescribed, but when it was, was associated with an adverse event risk 74% as high as rosuvastatin across all categories.

**Table 1:** Adverse Event Categories.

Myalgia (AEs searched: “myalgia”. 23,133 total cases* in AERS.)							
Drug Name	Primary AEs	All AEs	PR	Primary AEs/PR	All AEs/PR	Ranked Risk (Primary)	Ranked Risk (All)
Rosuvastatin	1,641	2,019	0.2901	5,657	6,959	100	100
Fluvastatin	103	184	0.0265	3,887	6,943	69	100
Atorvastatin	2,751	3,667	0.8604	3,197	4,262	57	61
Pravastatin	257	541	0.2275	1,130	2,378	20	34
Simvastatin	1,003	1,827	1	1,003	1,827	18	26
Lovastatin	67	189	0.2153	311	878	5	13

Myopathy (AEs searched: “myopathy”. 1,419 total cases* in AERS.)							
Drug Name	Primary AEs	All AEs	PR	Primary AEs/PR	All AEs/PR	Ranked Risk (Primary)	Ranked Risk (All)
Fluvastatin	9	15	0.0265	340	567	100	100
Rosuvastatin	77	102	0.2901	265	352	78	62
Atorvastatin	195	263	0.8604	227	306	67	54
Simvastatin	184	280	1	184	280	54	49
Lovastatin	15	21	0.2153	70	98	21	17
Pravastatin	13	44	0.2275	57	193	17	34

Myositis (AEs searched: “myositis”. 1,305 total cases* in AERS.)							
Drug Name	Primary AEs	All AEs	PR	Primary AEs/PR	All AEs/PR	Ranked Risk (Primary)	Ranked Risk (All)
Simvastatin	208	269	1	208	269	100	89
Fluvastatin	5	8	0.0265	189	302	91	100
Rosuvastatin	49	56	0.2901	169	193	81	64
Atorvastatin	112	159	0.8604	130	185	63	61
Pravastatin	12	25	0.2275	53	110	25	36
Lovastatin	3	5	0.2153	14	23	7	8

Rhabdomyolysis (AE searched: “rhabdomyolysis”. 8,111 total cases* in AERS.)							
Drug Name	Primary AEs	All AEs	PR	Primary AEs/PR	All AEs/PR	Ranked Risk (Primary)	Ranked Risk (All)
Fluvastatin	53	111	0.0265	2,000	4,189	100	100
Rosuvastatin	526	620	0.2901	1,813	2,137	91	51
Simvastatin	1,421	1,974	1	1,421	1,974	71	47
Atorvastatin	657	984	0.8604	764	1,144	38	27
Pravastatin	74	193	0.2275	325	848	16	20
Lovastatin	59	102	0.2153	274	474	14	11

Joints and Tendons (AEs searched: *please see File S2*. 53,168 total cases* in AERS.)							
Drug Name	Primary AEs	All AEs	PR	Primary AEs/PR	All AEs/PR	Ranked Risk (Primary)	Ranked Risk (All)
Rosuvastatin	746	1,239	0.2901	2,572	4,271	100	78
Fluvastatin	41	146	0.0265	1,547	5,509	60	100
Atorvastatin	1,241	2,890	0.8604	1,442	3,359	56	61
Simvastatin	470	1,871	1	470	1,871	18	34
Pravastatin	104	561	0.2275	457	2,466	18	45
Lovastatin	33	280	0.2153	153	1,301	6	24

Muscle Atrophy and Injury (AEs searched: *please see Supplement S2*. 3,949 total cases* in AERS.)							
Drug Name	Primary AEs	All AEs	PR	Primary AEs/PR	All AEs/PR	Ranked Risk (Primary)	Ranked Risk (All)
Fluvastatin	17	31	0.0265	642	1,170	100	100
Atorvastatin	405	554	0.8604	471	644	73	55
Rosuvastatin	128	174	0.2901	441	600	69	51
Pravastatin	46	111	0.2275	202	488	31	42
Simvastatin	154	295	1	154	295	24	25
Lovastatin	8	26	0.2153	37	121	6	10

Muscle Coordination and Weakness (AEs searched: *please see File S2*. 166,075 total cases* in AERS.)							
Drug Name	Primary AEs	All AEs	PR	Primary AEs/PR	All AEs/PR	Ranked Risk (Primary)	Ranked Risk (All)
Rosuvastatin	2,386	3,861	0.2901	8,225	13,308	100	81
Fluvastatin	148	434	0.0265	5,585	16,377	68	100
Atorvastatin	3,635	8,621	0.8604	4,225	10,020	51	61
Simvastatin	1,483	6,608	1	1,483	6,608	18	40
Pravastatin	256	1,693	0.2275	1,125	7,441	14	45
Lovastatin	123	798	0.2153	571	3,707	7	23

AERS – Adverse Events Reporting System; AEs – Adverse Events; PR – Prescribing Ratio.

* “total cases” for each item refers to the number of cases found in the AERS database using the respective search term.

A modest suggestion of higher reporting rates associated with rosuvastatin's market introduction cannot be clearly distinguished from higher adverse effect reporting for statins in general in 2004 and 5, relative to a nadir in 2006–8, then a rise again in 2009–10 (**Table 2**).

**Table 2:** Yearly Primary Suspect Case Reports by Outcome.

Rhabdomyolysis								
	2004	2005	2006	2007	2008	2009	2010	2011
Simvastatin	294	263	267	206	191	289	301	283
Atorvastatin	255	189	103	131	76	64	139	76
Rosuvastatin	114	135	80	80	90	113	102	104
Lovastatin	21	12	8	15	16	13	2	8
Pravastatin	21	19	14	9	13	12	10	13
Fluvastatin	22	16	8	7	11	7	10	3

Myalgia								
	2004	2005	2006	2007	2008	2009	2010	2011
Simvastatin	200	224	160	126	174	196	205	271
Atorvastatin	293	308	321	396	259	199	1,283	415
Rosuvastatin	356	208	285	172	308	460	372	324
Lovastatin	10	14	7	17	4	17	11	8
Pravastatin	68	67	14	53	36	45	42	28
Fluvastatin	28	45	24	21	13	11	7	6

Myopathy								
	2004	2005	2006	2007	2008	2009	2010	2011
Simvastatin	53	37	23	32	23	49	36	63
Atorvastatin	39	41	42	39	19	20	48	18
Rosuvastatin	23	19	15	20	7	14	14	16
Lovastatin	2	1	2	5	3	4	1	0
Pravastatin	3	2	2	0	2	6	1	1
Fluvastatin	3	4	1	1	2	2	1	1

Myositis								
	2004	2005	2006	2007	2008	2009	2010	2011
Simvastatin	47	34	22	25	38	37	62	46
Atorvastatin	37	26	25	21	12	11	26	17
Rosuvastatin	28	16	12	10	4	11	6	5
Lovastatin	2	2	0	1	1	1	0	1
Pravastatin	7	6	2	2	2	1	0	0
Fluvastatin	6	1	2	0	2	0	0	1

Nausea (non-muscle comparator)								
	2004	2005	2006	2007	2008	2009	2010	2011
Simvastatin	30	44	33	33	40	44	48	35
Atorvastatin	62	64	38	62	32	35	131	52
Rosuvastatin	121	59	77	34	86	124	102	88
Lovastatin	2	1	3	4	0	2	2	1
Pravastatin	14	6	3	6	8	14	11	10
Fluvastatin	13	10	5	11	2	5	0	1

**Table S1** represents the number of specific unfavorable outcomes (“death,” “disability,” “hospitalization - initial or prolonged,” “life-threatening,” and “required intervention to prevent permanent impairment/damage”) associated with myalgia, myopathy, myositis, and rhabdomyolysis for each drug. The final four tables represent total case report counts for each of the above outcome measures from the combination of myalgia, myopathy, myositis, and rhabdomyolysis. The majority of reports for the more serious adverse events, such as myositis and rhabdomyolysis, are generated by healthcare professionals while consumers account for higher reporting percentages for what are commonly deemed less serious side effect categories such as myalgia, or “joints and tendons” (**Table S2**).

## Discussion

Among commonly prescribed statins, rosuvastatin appears to be linked to the highest adverse event risks reported across most muscle-related side effect categories in post-marketing patient populations. Lovastatin and pravastatin appeared to have the lowest risk rates. These findings, based on a significant volume of case reports in the FDA AERS database linking statins with muscle adverse effects, corroborate and extend existing knowledge regarding the association of statin drugs with muscle-related adverse events. Our results parallel those of Sakaeda et al., 2011 [14], Cham et al., 2010 [5], and Alsheikh-Ali et al., 2005 [15]. Additionally, the findings generally corroborate those of Cham et al., 2010 in observing that fluvastatin, an agent that was not commonly prescribed, was an apparent potency outlier [5]. Using a patient-targeted survey approach, they demonstrated that: (i) the highest potency statins (rosuvastatin and atorvastatin) showed higher muscle adverse event rates, (ii) simvastatin, with intermediate potency, showed intermediate rates, and finally (iii) pravastatin and lovastatin, with their lower potencies, showed the lowest rates [5]. (See **Table S3** for relative dose equivalence of statins.) We think a likely reason is that fluvastatin, which is far less frequently prescribed than other statins, may be primarily reserved by physicians for patients who have failed to tolerate other statins. Disproportionate use in statin non-tolerators may produce higher apparent adverse effect rates (indeed, use of fluvastatin 80 mg for those intolerant to other statins is advised by some [6]). It might also be selectively used in settings in which drug interactions or other factors heighten toxicity. Alternatively, of course, fluvastatin might actually engender risk of muscle adverse effects beyond expectation for its potency. Head-to-head randomized assessments of fluvastatin versus other agents (or within-person crossover comparisons like the potency comparisons of Cham et al. [5]) are desirable to resolve this.

Sakaeda et al., 2011 [14] analyzed the AERS database using methods similar to ours and found that muscle-related adverse events were more commonly observed with rosuvastatin treatment when compared with other statins such as atorvastatin and pravastatin. However, their inferences differ from our own regarding the foundation for such differences, as they do not ascribe a primary role to statin potency [14]. Alsheikh-Ali et al., 2005 analyzed the first year of rosuvastatin AERS data against other major statins with a “first year of marketing analysis” and a “concurrent time period analysis” [15]. Corresponding to our findings, their analysis showed that rosuvastatin had a higher risk rate for important muscle-related side effects.

Other studies that have analyzed post-marketing adverse events linked to rosuvastatin include Wolfe and Zipes et al. [16], [17]. Both used data taken from the small time window of approximately one year following rosuvastatin's introduction into the US market. In contrast to our findings and those cited above, the Zipes' et al., 2006 study appeared to show no difference in risk rates between rosuvastatin and other statins [17]. However, that study used the ratio of a given adverse event report to all adverse event reports for the drug as the index. This approach may preclude detection of even large increases in adverse events if they are proportional (all adverse events increased together), as might arise from factors like greater potency. The Wolfe 2004 paper cited high renal and muscle-related side effect rates from both pre- and post-marketing data [16]. Wolfe specifically focused on potentially high rhabdomyolysis risks in calling for rosuvastatin to be pulled from the market [16].

From a pharmacokinetic perspective (without consideration of potency), rosuvastatin has a profile that might be expected to yield fewer, not more, adverse events. It has high hepatoselectivity, high hydrophilicity, low rates of metabolism via cytochrome P450 enzymes, and moderate systemic bioavailability [18], [19]. Typical statements in the scientific literature appear to suggest rosuvastatin's safety equals or surpasses that of other statins, with assertions that rosuvastatin: is “safe and well tolerated,” [20] has a “safety profile comparable to other statins” [21], “has a superior safety profile,” [22] or even “an improved clinical safety profile” [23].

Our data suggest, however, that any benefits attending such factors may be overridden by other factors, such as potency considerations. Higher potency agents, and rosuvastatin in particular, were associated with elevated relative risk of adverse events. This finding has important implications for statin treatment decisions in general, and particularly with regard to patients who have already experienced muscle-related adverse events from statin therapy.

The parallels between our results and findings from prior adverse event surveys corroborate and validate the idea that valuable information can be obtained from within-class drug comparisons based solely upon AERS data. For key purposes, then, such an approach may lessen the need for, and costs associated with, separate adverse event surveys targeting individual drug classes.

This is important because of the central role of postmarketing information in the assessment of drug safety. Randomized clinical trials (RCTs) are considered the highest quality evidence for causal inference, but they have significant limitations for adverse effect detection and analysis. These include methods that exclude subjects who may have polypharmacy risks, are elderly, have comorbidities, and known risk factors for harm. Such patient-selection practices differentially exclude precisely those subjects who might be most likely to experience adverse events. Additional factors that can limit clinical trial utility for adverse effect understanding include: (i) exclusions based on statin compliance [24] (lower compliance is linked to statin adverse effects [25]), (ii) restrictive definitions for “statin myopathy” (e.g. requiring CK elevations >10× ULN), (iii) drug exposure times that are short relative to those experienced in many post-marketing consumer populations, (iv) treatment discontinuation at the first sign of a problem (yielding different findings than real-world usage), and (v) lack of comparator data across statin agents.

The relationship noted here between reported muscle-related adverse effects and statin potency (in terms of LDL reduction) does not necessarily imply that muscle adverse events are caused by lowering LDL cholesterol. The potency of LDL reduction relates to the magnitude of mevalonate inhibition, which, in turn, affects coenzyme Q10 levels, testosterone reduction in men, reduced antioxidant transport, and numerous other factors.

Postmarketing surveillance bears well-recognized limitations, such as lack of randomization, and is not intended to replace RCT approaches. Nonetheless, for the reasons noted above, RCTs are disadvantaged in adverse event detection, and case reports and postmarketing surveillance are commonly responsible for the first identification of important adverse events, including those that ultimately lead to regulatory actions such as “black box” warnings and product withdrawals [26], [27]. Each approach has an important role, and complements the other to extend the understanding of drug benefits versus risks. Limitations of our analysis include: (i) the FDA AERS database is only as accurate as the information inputted into it from various sources. (ii) AERS does not filter, correct, or make any analysis of the quality or potential bias of inputted data. (iii) Exogenous factors such as publicity and marketing can influence reporting. (iv) Physicians might disproportionately report effects associated with newer drugs, and rosuvastatin is the newest of the statins studied. Our analysis, however, did not find clear support for this limitation. (v) Dose data are not available. Physicians could prescribe higher doses of one statin within the recommended dosage range. However, maximum potencies of use bear an expected relation to average potencies of use. Moreover, in the Cham analysis of adverse effects, consistent results were obtained whether looking at results by expected potency equivalencies without consideration of dose and when evaluating individual rechallenge cases with known drug and dose. Both presumed potency for the statin, and known potency based on dose and dose equivalencies, were predictors of relative adverse event rates [5]. (vi) Reports submitted to the FDA contain mistakes, including spelling errors leading to misclassifications, important data either missing or inadequately reported, and duplicate reports; however our analysis systems included multiple processing steps, safeguards, and manual oversight to lessen the impact of such factors. (vii) Only a minority of post-marketing adverse events are believed to be successfully logged into AERS [28]. Therefore, any calculated rates are apt to substantially underestimate the actual incidence of these side effects in broad consumer populations. We address this by use of comparative rates.

Given that: (i) relative adverse event risks appear to be higher with higher potency statins, and (ii) other data sources (meta-analyses of head to head statin trials) have demonstrated that mortality outcomes are not more favorable with higher potency statin use (except in acute coronary syndrome) [29], we suggest these findings favor use of lower potency statins (if statin use is clearly indicated), particularly where a previous statin myopathy has occurred.

The data presented in this report may offer important reference points regarding the selection of statins for cholesterol management in general, and especially for the rechallenge of patients that have experienced muscle-related side effects. *If* statin reinitiation is considered following muscle-related adverse effects, agents of lower expected potency should be preferred. We believe that our results warrant the attention of healthcare providers, drug developers, patients, and regulatory professionals involved with statins and other cholesterol-related medications. Moreover, the data mining approach employed appears promising, and may have application in evaluation of adverse reactions from other drug classes.

## Supporting Information

File S1 **FDA AERS – Food and Drug Administration Adverse Events Reporting System.** This listing includes the names by which each drug is known as well as any misspellings that were identified.
(DOC)

File S2 **AERS – Adverse Events Reporting System.** This file S2 delineates AERS search terms used for each adverse event category.
(DOC)

Table S1 **AEs – Adverse Events.** For each muscle adverse event category this table lists the number of major clinical outcomes (e.g., death, disability, hospitalization) associated with that outcome, for each statin agent.
(DOC)

Table S2 **This shows the percent contribution to statin adverse event reports by different groups (e.g., physician, pharmacist, consumer), stratified by adverse event category and statin drug.**
(DOC)

Table S3 *Based on similar LDL reduction [**30]**, [**31**] **.** Note: These are inexact equivalency approximations.
(DOC)
