# Supplementary material for: A Survey of the FDA's AERS Database Regarding Muscle and Tendon Adverse Events Linked to the Statin Drug Class
Source: PLoS One. 2012 Aug 22;7(8):e42866. doi: 10.1371/journal.pone.0042866 (PMC3425581; doi:10.1371/journal.pone.0042866)
Supplement: File S1 — FDA AERS – Food and Drug Administration Adverse Events Reporting System. This listing includes the names by which each drug is known as well as any misspellings that were identified. (DOC) [file pone.0042866.s001.doc]

**Supplement S1**

Separate Designations Listed in the FDA AERS database, Aggregated into a Single Name.

Rosuvastatin

FDA Listed Names (248):

Cerstor, Creastor, Creator (rosuvastatin Calcium), Creator (rosuvastatin), Cresotr /01588601/ (rosuvastatin Sodium), Crestar (rosuvastatin), Crester, Crester /01588601/, Crestior, Crestir, Crestir (rosuvastatin Calcium), Cresto, Crestol, Creston, Creston (all Other Therapeutic Products), Creston (rosuvastatin Calcium), Creston(rosuvastatin Calcium), Crestor, Crestor /01588601/, Crestor /01588602/, Crestor /01588601/ (rosuvastatin), Crestor (rosuvastatin Calcium) (5 Milligram), Crestor (rosuvastatin), 10 Mg, Crestor /net/(rosuvastatin Calcium), Crestor (rosuvasatin), Crestor /01588801/ (rosuvastatin), Crestor 20 Mg. Astra-zenca, Crestor Astrazeneca, Crestor (10 Mg, Tablet) (rosuvastatin), Crestor (5 Milligram), Crestor (all Other Therapeutic Products), Crestor (all Other Therapeutoc Products), Crestor (all Therapeutic Products) Tablets, Crestor (cholesterol), Crestor (cholesterol- And Triglyceride Reducers), Crestor (cholesterol- And Triglyceride Reducers) Tablets, Crestor (cholesterol- And Triglycerides Reducers), Crestor (con.), Crestor (crestor), Crestor (rosovastatin), Crestor (rosovastatin)(rosuvastatin), Crestor (rosuavstatin Calcium), Crestor (rosucastatin), Crestor (rosulvastatin Calcium), Crestor (rosumvastatin Calcium), Crestor (rosusvastatin), Crestor (rosuvasatin), Crestor (rosuvastain Calcium) (rosuvastatin Calcium), Crestor (rosuvastain) (rosuvastatin), Crestor (rosuvastatain), Crestor (rosuvastatin Calciuim), Crestor (rosuvastatin Calcium (tablet) (rosuvastatin Calcium), Crestor (rosuvastatin Calcium), Crestor (rosuvastatin Calcium) I, Crestor (rosuvastatin Calcium) (10 Milligram, Tablets) (rosuvastatin S, Crestor (rosuvastatin Calcium) (20 Milligram), Crestor (rosuvastatin Calcium) (tablet) (rosuvastatin Calcium), Crestor (rosuvastatin Calcium) (tablets), Crestor (rosuvastatin Calcium) (tablets) (rosuvastatin Calcium), Crestor (rosuvastatin Calcium) (unknown) (rosuvastatin Calcium), Crestor (rosuvastatin Calcium) 10 Mg, Crestor (rosuvastatin Calcium) Film-coated Tablet, Crestor (rosuvastatin Calcium) Film-coated Tablet, 10 Mg, Crestor (rosuvastatin Calcium) Formulation, Crestor (rosuvastatin Calcium) Per Oral Nos, Crestor (rosuvastatin Calcium) Unk To Ongoing, Crestor (rosuvastatin Calcium)(20 Milligram), Crestor (rosuvastatin Calicium), Crestor (rosuvastatin Calicum), Crestor (rosuvastatin Calsium), Crestor (rosuvastatin Clacium), Crestor (rosuvastatin Galcium), Crestor (rosuvastatin Sodium), Crestor (rosuvastatin Sodium) (rosuvastatin Sodium), Crestor (rosuvastatin), Crestor (rosuvastatin) ( To Unknown), Crestor (rosuvastatin) (10 Milligram) (rosuvastatin), Crestor (rosuvastatin) (10 Milligram, Tablets), Crestor (rosuvastatin) (20 Milligram, Tablets) (rosuvastatni), Crestor (rosuvastatin) (40 Milligram, Tablets), Crestor (rosuvastatin) (5 Milligram), Crestor (rosuvastatin) (75 Mg) (rosuvastatin), Crestor (rosuvastatin) (rosuvastatin Calcium), Crestor (rosuvastatin) (rosuvastatin), Crestor (rosuvastatin) (rosuvstatin), Crestor (rosuvastatin) (tablets), Crestor (rosuvastatin) (tablets) (rosuvastatin), Crestor (rosuvastatin) (unknown), Crestor (rosuvastatin) Ongoing, Crestor (rosuvastatin) Tablet, Crestor (rosuvastatin) Tablets, Crestor (rosuvastatin)(5 Milligram) (rosuvastatin), Crestor (rosuvastatin0 (rosuvastatin), Crestor (rosuvastatine Calcium), Crestor (rosuvastatine), Crestor (rosuvastattn Calctttmi (rorttvastattn Calcium), Crestor (rosuvastin), Crestor (rosuvastin) (rosuvastin), Crestor (rosuvatatin), Crestor (rosuvsatatin Calcium), Crestor (rosuvstatin), Crestor (rosvastatin Calcium), Crestor (rosvastatin Calcium) (tablets), Crestor (rosvastatin), Crestor (rosvuastatin Calcium), Crestor (rosvuastatin), Crestor (rousvastatin) Rosusvastatin), Crestor (rusuvastatin), Crestor /-1588601/, Crestor /0158601/, Crestor /01588601/ (con.), Crestor /01588601/ (crestor), Crestor /01588601/ (rosuvastatin), Crestor /01588601/ (unknown), Crestor /01588601/(rosuvastatin), Crestor /01588602, Crestor /01588602/, Crestor /01588602/ (con.), Crestor /01588602/ (rosuvastain Calcium), Crestor /net/ (rosuvastatin Calcium), Crestor /net/ (rosuvastatin Calcium), 20 Mg, Crestor /net/(rosuvastatin Calcium) Oral Single Dose, Crestor 10 Mg -to Be Cut In Half Glaco Kline-, Crestor 10 Mg Astra Zaneca, Crestor 10 Mg Astrazeneneca, Crestor 10 Mg. Astra Zenaca, Crestor 20 Mg Every Day, Ongoing, Crestor 20 Mg. Astra-zenca, Crestor Astrazeneca, Crestor Cr, Crestor Nos, Crestor Tablet, Crestor Tablets 5mg, Crestor(osuvastatin Calcium), Crestor(rosuvastatin) (tablets), Crestor(rosuvastatin)(20 Milligram, Tablets)(rosuvastatin), Crestor/ (rosuvasatatin Calcium), Crestor/01588601/ (rosuvastatin), Crestor/net/ (rosuvastatin Calcium), Crestor/net/(rosuvastatin Calcium), Crestpr, Crestpr (rosuvastatin), Crestro, Cretor (rosuvastatin Calcium) (rosuvastatin Calcium), Cristor, Crostor, Dilatrane /00012201/ (dilatrane Rosuvastatin) (not Specified), Provisacor, Rasuvhstatin Calcium, Resuvastatin, Rosevastin, Rosouvastatin, Rosulvastatin (crestor), Rosuva, Rosuvacard, Rosuvasatatin Calcium, Rosuvasatin, Rosuvast, Rosuvastain (crestor) Astra Zenica, Rosuvastastin Code Not Broken, Rosuvastatic, Rosuvastatin, Rosuvastatin (rosuvastatin), Rosuvastatin (rosuvastatin), Rosuvastatin (rosuvastatin), Rosuvastatin (crestor /01588601/), Rosuvastatin (rosuvastatin), Rosuvastatin 10 Mg Astrazeneca, Rosuvastatin 20 Mg, Rosuvastatin 5mg Aztra, Rosuvastatin 10 Mg Astra Zenea, Rosuvastatin 10mg Astrazeneca, Rosuvastatin 20 Mg, Rosuvastatin 5mg Astrazeneca, Rosuvastatin (rosuvastatin), Rosuvastatin 40 Mg, Rosuvastatin (all Other Therapeutic Products), Rosuvastatin (crestor / 01588601/), Rosuvastatin (crestor /01588601/), Rosuvastatin (crestor 01588601), Rosuvastatin (crestor) Hydrochlorothiazide, Rosuvastatin (raze), Rosuvastatin (rosuvastatin), Rosuvastatin (rosuvastatin) Tablet, 10mg, Rosuvastatin (rosuvastatin) (rosuvastatin), Rosuvastatin (rosuvastatin) Tablet, 10mg, Rosuvastatin (rosuvatatin) (rosuvastatin), Rosuvastatin (rousvastatin), Rosuvastatin (suspect), Rosuvastatin (tablets), Rosuvastatin (unknown), Rosuvastatin 10 Mg, Rosuvastatin 10 Mg (astrazeneca), Rosuvastatin 10 Mg Astra Zenea, Rosuvastatin 10 Mg Astra-zeneca, Rosuvastatin 10 Mg Astrazeneca, Rosuvastatin 10 Mg Tab Astra/zeneca, Rosuvastatin 10mg, Rosuvastatin 10mg (astrazeneca), Rosuvastatin 10mg Astrazeneca, Rosuvastatin 20 Mg, Rosuvastatin 20 Mg Astrazeneca, Rosuvastatin 40 Mg, Rosuvastatin 40 Mg Astra-zeneca, Rosuvastatin 40mg, Rosuvastatin 5 Mg, Rosuvastatin 5mg Astrazeneca, Rosuvastatin 9fosuvastatin) (rosuvastatin), Rosuvastatin 9rosuvastatin), Rosuvastatin Ca, Rosuvastatin Calcium, Rosuvastatin Calcium (crestor), Rosuvastatin Calcium (formulation Unknown) (rosuvastatin Calcium), Rosuvastatin Calcium (rosuvastatin Calcium), Rosuvastatin Calcium (rosuvastatin), Rosuvastatin Calcium And Rosuvastatin Sodium, Rosuvastatin Calctum (rosuvastatin Calcium), Rosuvastatin Calsium, Rosuvastatin Code Not Broken, Rosuvastatin Or Placebo, Rosuvastatin Sodium, Rosuvastatin(rosuvastatin), Rosuvastatin-10mg, Rosuvastatin-calcium, Rosuvastatin, Crestor, Astrazeneca, Antilipemic, Rosuvastatina, Rosuvastatine, Rosuvastatine (rosuvastatin Calcium) (tablets) (rosuvastatin Calcium), Rosuvastatine (rosuvastatin), Rosuvastatine (rosuvastatine), Rosuvastatine (tablets), Rosuvastation, Rosuvastatn, Rosuvasterine, Rosuvastine, Rosuvastine(crestor), Rosuvasttin Calcium, Rosuvostatine, Rosuvustatin, Rosvastatin, Rouvastatin, Roxuvastatina, Rozavel, Rusovastatin, Simestat, Tab Rosuvastatin Calcium 10 Mg, Unspecified, Visacor

Fluvastatin

FDA Listed Names (201):

(fractal), Canef, Canef (lic. Astra), Canef (licensed To Astra), Cranoc, Cranoc (fluvastatin Sodium), Cranoc (fluvastatin Sodium), Cranoc (licensed, Astra), Cranoc (licensed, Fujisawa Deutschland), Cranoc 40, Cranoc 80, Cranoc 80 Retard, Digaril, Digaril Prolib, Digaril Prolib (fluvastatin Sodium) (80 Milligram) (fluvastatin Sodium, Flovastatin, Fluovastatin (fluovastatin), Fluovastatin Sodium, Fluvasatin, Fluvastatin, Fluvastatin /01224501/, Fluvastatin (fluvastatin), Fluvastatin (fluvastatin), Fluvastatin (fluvastatin), Fluvastatin (fluvastatin), Fluvastatin (fluvastatin), Fluvastatin 40mg, Fluvastatin (fluvastatin), Fluvastatin (fluvastatin /01224501/), Fluvastatin (fluvastatin), Fluvastatin (fluvastatin) (kah), Fluvastatin (fluvastatin) Unknown, Fluvastatin (lescol) (fluvastatin), Fluvastatin (ngx), Fluvastatin (ngx) (fluvastatin), Fluvastatin /01224502/, Fluvastatin 20 Mg, Fluvastatin 40mg, Fluvastatin 40mg Cap, Fluvastatin 80 Mg, Fluvastatin 80mg Xl, Fluvastatin Beta, Fluvastatin G-fluv+xrtab, Fluvastatin Na, Fluvastatin Na (lescol), Fluvastatin Sa 80 Mg, Fluvastatin Sodiuim (fluvastatin Sodium), Fluvastatin Sodium, Fluvastatin Sodium (fluvastatin Sodium), Fluvastatin Sodium (fluvastatin Sodium), Fluvastatin Sodium (fluvastatin Sodium), Fluvastatin Sodium (con.), Fluvastatin Sodium (fluvastatin Sodium), Fluvastatin Sodium (fluvastatin Sodium) Tablets, Fluvastatin Sodium (lescol), Fluvastatin Sodium (lescol) Capsules, Fluvastatin Sodium (lochol), Fluvastatin Sodium 20mg Cap, Fluvastatin Sodium Capsule, Fluvastatin Sodium Sa (lescol Xl) Stablel, Fluvastatin Sodium Sa (lescol Xl) Stablet, Fluvastatin Sodium Tablet (fluvastatin Sodium), Fluvastatin Sodium), Fluvastatin Soduim (fluvastatim Sodium), Fluvastatin Soidum, Fluvastatin Tablets, Fluvastatin Vs Acetaminophen Vs Placebo, Fluvastatin Xl, Fluvastatin(fluvastatin), Fluvastatin-lescol, Fluvastatina, Fluvastatine, Fluvistatin, Fluvstatin (fluvastatin), Fractal, Fractal /01224501/, Fractal /01224501/, Fractal (flurvastatin), Fractal (fluvastatin), Fractal (fluvastatin,), Fractal (fluvastatine), Fractal (fluvsastatin), Fractal (pluvastatin), Fractal 20, Fractal Lp, Fulvistatin, Ldescol (fluvastatin) (40 Milligram), Lesacol, Leschol, Leschol (fluvastatin Sodium) Tablets, Lesco Xl, Lescol, Lescol /01224501/, Lescol /01224501/, Lescol (80 Mg, Capsule) (fluvastatin), Lescol (capsule (fluvastatin Sodium), Lescol (con.), Lescol (flouvastatin Sodium), Lescol (fluvastatin /01224501/), Lescol (fluvastatin /01224501/), Lescol (fluvastatin /01224501/), Lescol (fluvastatin /01224501/), Lescol (fluvastatin /012245501/), Lescol (fluvastatin Sodium) (capsules) (fluvastatin Sodium), Lescol (fluvastatin Sodum), Lescol (fluvastatin) Coated Tablet, Lescol (fluvastatine Sodium), Lescol (fluvastatinn Sodium), Lescol (fluvastatn Sodium), Lescol (fluvastin), Lescol (fluvstatin Sodium), Lescol (fulvastatin), Lescol (tablets) Fluvastatin Sodium), Lescol - Cholesterol, Lescol /0122450/ (fluvastatin), Lescol /01224501/, Lescol /01224501/ (fluvastatin), Lescol /01224501/(fluvastatin), Lescol /01224502/, Lescol /01224502/ (fluvastatin Sodium), Lescol /sch/, Lescol 20 Mg (fluvastatin Sodium), Lescol 40, Lescol Capsule, Lescol Forte, Lescol Hazzard, Lescol Les+, Lescol Les+cap, Lescol Les+srf, Lescol Les+tab, Lescol Lp, Lescol Lp (fluvastatin), Lescol Lp (fluvastatin Sodium), Lescol Mr, Lescol Mr (fluvastatin), Lescol Mr Les01+, Lescol Retard (fluvastatin), Lescol Xl, Lescol Xl (fluvastatin Sodium Extended-release Tablets), Lescol Xl (fluvastatin Sodium) (tablets), Lescol Xr, Lescol Xr (fluvastatin Sodium), Lescol ^norvartis^, Lescol ^novartis^ (fluvastatin Sodium), Lescol ^novartis^(fluvastatin Sodium), Lescol ^sandoz^, Lescol ^sandoz^ (fluvastatin Sodium), Lescol-xl (fluvastatin), Lescol^novartis^ (fluvastatin Sodium), Lipaxan, Lipaxan (italfarmaco), Liposit Prolib, Local (fluvastatin Sodium), Lochol, Lochol /00638501/, Lochol /01224501/, Lochol /01224502/, Lochol (fluvastatin Sodium), Lochol (fluvastatin Sodium), Lochol (fluvastastin) Tablet, Lochol (fluvastatin Sdoium), Lochol (fluvastatin Sodium, Lochol (fluvastatin Sodium ) (ta), Lochol (fluvastatin Sodium), Lochol (fluvastatin Sodium) (fluvastatin Sodium), Lochol (fluvastatin Sodium) (ka), Lochol (fluvastatin Sodium) (nr), Lochol (fluvastatin Sodium) (ta), Lochol (fluvastatin Sodium) (tablets), Lochol (fluvastatin Sodium) Tablet, Lochol (fluvastatin Sodium)(tablet) (fluvastatin Sodium), Lochol (fluvastatin Soidum), Lochol (fluvastatin) Tablet, Lochol (japan), Lochol /01224502/ (lochol), Lochol /01224502/), Lochol Per Oral Nos, Lochol Tablet, Lochol(fluvastatin Sodium), Locol, Locol (fluvastatin Sodium), Locol (fluvastatin Sodium), Locol (fluvastatin Sodium) Tablet, Locol (fluvastatin Sodium), Locol (fluvastatin Sodium) (20 Mg), Locol (fluvastatin Sodium) 80mg, Locol (fluvastatn Sodium), Locol / Xu, Locol 20 (fluvastatin Sodium), Locol 40, Locol 80, Locol Per Oral Nos, Locol Retard, Locol Tablet, Locol? (fluvastatin Sodium), Lsecol, Lsecol (fluvastatin), Lymetel, Lymetel (lic To Laboratorios Andromaco), Lymetel Prolib (lic To Laboratorios Andromaco), Primesin

Atorvastatin

FDA Listed Names (810):

(atoravastatin), (atorvastatin), (atorvastatin) - Tablet - 40 Mg, (atorvastttin), *atorvastatin, ,ipitor, ,ipitor (atorvastatin Calcium), Actorvastatin (atorvastatin), Aorvastatin, Apo-atorvastatin, Apo-atrovastatin (atrovastatin Caclium) (atrovastatin Calcium), Artovastatin, Aspavor, Aspavor (atorvastatin Calcium), Astorvastatin, Astorvastatin (atorvastatin), Astorvastatin -lipitor-, Atoravastatin, Atoravastatin Calcium, Atoravastatin Calcium (lipitor), Atoravstatin (atorvastatin), Atorbvastatin, Atoris, Atoris /01326101/, Atoris (atorvastatin Calcium), Atoris (atorvastatin), Atoris (atorvastatin) Unspecified, Atorlip, Atorva, Atorva Teva / Atorvastatin, Atorvas, Atorvasatatin, Atorvasatin, Atorvasatin (atorvastatin), Atorvasatine, Atorvasstatin, Atorvastaatin Calcium, Atorvastaatin Calcium (ljpitor), Atorvastain, Atorvastain ( Atoravstatin), Atorvastain (atorvastatin), Atorvastain Calcium, Atorvastaine, Atorvastan, Atorvastati (atorvastatin), Atorvastatien, Atorvastatin, Atorvastatin (atorvastatin), Atorvastatin (atorvastatin), Atorvastatin /01326102/, Atorvastatin (atorvastatin), Atorvastatin (atorvastatin), Atorvastatin (sortis), Atorvastatin 80mg/placebo, Atorvastatin (atorvastatin), Atorvastatin (atorvastatin), Atorvastatin - Tablet - 40 Mg, Atorvastatin (atorvasatin), Atorvastatin (atorvastatin), Atorvastatin (atorvastatin), Atorvastatin (atorvastatin), Atorvastatin /01326102/ (atorvastatin Calcium), Atorvastatin (atorvastatin), Atorvastatin (atorvastatin), Atorvastatin (atorvastatin), Atorvastatin (atorvastatin Calcium) (unknown), Atorvastatin (atorvastatin), Atorvastatin (atorvastatin), Atorvastatin (atorvastatin), Atorvastatin 20 Mg Tablets, Atorvastatin 20 Mg Pfizer, Atorvastatin (atorvastatin), Atorvastatin 10mg Pfizer, Atorvastatin 40, Atorvastatin 80 Mg, Atorvastatin 80mg Pfizer, Atorvastatin Parke-davis, Atorvastatin (atorvastatin), Atorvastatin ( Atorvastatin), Atorvastatin (10 Mg), Atorvastatin (10 Milligram, Tablets), Atorvastatin (aotrvastatin), Atorvastatin (atorastatin), Atorvastatin (atorasvastatin), Atorvastatin (atoravastatin) (atorvastatin), Atorvastatin (atorvasatatin), Atorvastatin (atorvasatin), Atorvastatin (atorvastain), Atorvastatin (atorvastain, 10mg, Discontinued After Adverse Event), Atorvastatin (atorvastastin), Atorvastatin (atorvastati N), Atorvastatin (atorvastatin ), Atorvastatin (atorvastatin Calcijm), Atorvastatin (atorvastatin Calcium), Atorvastatin (atorvastatin Calcium) (atorvastatin Calcium), Atorvastatin (atorvastatin Calcium) (atorvastatin), Atorvastatin (atorvastatin Calcium)(tablets)(atorvastatin Calcium), Atorvastatin (atorvastatin Calicum), Atorvastatin (atorvastatin), Atorvastatin (atorvastatin) (20 Milligram, Tablet) (atorvastatin), Atorvastatin (atorvastatin) (40 Mg), Atorvastatin (atorvastatin) (atorvastatin), Atorvastatin (atorvastatin) (con.), Atorvastatin (atorvastatin) (nr), Atorvastatin (atorvastatin) (ta), Atorvastatin (atorvastatin) (tablet) (atorvastatin), Atorvastatin (atorvastatin) (tablets) (atorvastatin), Atorvastatin (atorvastatin) 10 Mg, Atorvastatin (atorvastatin) 1dose Form, Atorvastatin (atorvastatin) 20 Mg, Atorvastatin (atorvastatin) 20mg, Atorvastatin (atorvastatin) Coated Tablet, Atorvastatin (atorvastatin) Syrup, Atorvastatin (atorvastatin) Tablets, Atorvastatin (atorvastatin) Unknown, Atorvastatin (atorvastatin) Unspecified, Atorvastatin (atorvastatin) [10005747 - Blood Pressure High] [v.14.1], Atorvastatin (atorvastatin)(atorvastatin), Atorvastatin (atorvastatin, , 0), Atorvastatin (atorvastatin0, Atorvastatin (atorvastatin0 (atorvastatin), Atorvastatin (atorvastatine), Atorvastatin (atorvastation), Atorvastatin (atorvastatn), Atorvastatin (atorvastin), Atorvastatin (atorvasttin), Atorvastatin (atorvatatin), Atorvastatin (atovastatin), Atorvastatin (atrocastatin), Atorvastatin (blinded), Atorvastatin (con.), Atorvastatin (con.) /01326102/, Atorvastatin (lipitor Atorvastatin), Atorvastatin (ngx), Atorvastatin (ngx) (atorvastatin), Atorvastatin (ngx)(atorvastain) Unknown, Atorvastatin (no Pref. Name), Atorvastatin (see Attached Pages For Additional Suspect Drugs), Atorvastatin (sortis), Atorvastatin (tahor), Atorvastatin (unknown), Atorvastatin - Generic Lipitor, Atorvastatin - Nf*, Atorvastatin - Pfizer 10 Mg, Atorvastatin - Tablet - Unit Dose : Unknown, Atorvastatin - Tablet - Unit Dose: Unknown, Atorvastatin -generic Lipitor-, Atorvastatin -generic-, Atorvastatin -lipitor(r)-, Atorvastatin /01326101/ (atorvastatin), Atorvastatin /01326102/ (atorvastatin Calcium), Atorvastatin 10 Mg Parke-davis, Atorvastatin 10mg Pfizer, Atorvastatin 10mg Tablets (pfizer), Atorvastatin 20 Mg (mfr. Ni), Atorvastatin 20 Mg Merck, Atorvastatin 20 Mg Pfizer, Atorvastatin 20 Mg Tablets, Atorvastatin 20 Mg. Parke Davis, Atorvastatin 20mg Lipitor, Atorvastatin 20mg Tab, Atorvastatin 20mg Tablet (lipitor, Pfizer), Atorvastatin 40, Atorvastatin 40 Mg Coated Tablets, Atorvastatin 40 Mg Pfizer, Atorvastatin 40mg / 20mg, Coated Tablets (atorvastatin), Atorvastatin 40mg, Coated Tablets (atorvastatin), Atorvastatin 80 Mg, Atorvastatin 80 Mg Daily, Atorvastatin 80mg, Atorvastatin 80mg (hypercholesterolaemia), Atorvastatin 9atorvastatin), Atorvastatin 9atorvastatin) (con.), Atorvastatin 9atorvastatin0, Atorvastatin A (atorvastatin), Atorvastatin Besilate, Atorvastatin Caclcium, Atorvastatin Caclium, Atorvastatin Cal, Atorvastatin Calcium, Atorvastatin Calcium (lipitor), Atorvastatin Calcium (lipitor), Atorvastatin Calcium (lipitor), Atorvastatin Calcium (lipitor), Atorvastatin Calcium (lipitor), Atorvastatin Calcium (atorvastatin Calcium) Tablet, Atorvastatin Calcium (20 Mg), Atorvastatin Calcium (atorvastatin Calcium) (atorvastatin Calcium), Atorvastatin Calcium (atorvastatin Calcium) (tablets) (atorvastatin Ca, Atorvastatin Calcium (atorvastatin Calcium) (unknown), Atorvastatin Calcium (atorvastatin Calcium) Tablet, Atorvastatin Calcium (atorvastatin Calcium) Unknown, Atorvastatin Calcium (con.), Atorvastatin Calcium (formulation Unknown) (generic) (atorvastatin Cal, Atorvastatin Calcium (lipitor), Atorvastatin Calcium (lipitor) (atorvastatin Calcium), Atorvastatin Calcium (liptor), Atorvastatin Calcium (sortis), Atorvastatin Calcium (tahor), Atorvastatin Calcium (to Unknown), Atorvastatin Calcium Film-coated Tablet, Atorvastatin Calcium Hydrate, Atorvastatin Calcium Hydrate (atorvastatin Calcium ), Atorvastatin Calcium Hydrate (atorvastatin Calcium Hydrate), Atorvastatin Calcium Hydrate (atorvastatin Calcium Hydrate) Tablet, Atorvastatin Calcium Hydrate (atorvastatin Calcium), Atorvastatin Calcium Oral, Not Otherwise Specified, Atorvastatin Calcium Tablets, Atorvastatin Calclium, Atorvastatin Clacium (atorvastatin Calcium), Atorvastatin Family, Atorvastatin Film-coated Tablet, Atorvastatin Oral Drug Unspecified Form, Atorvastatin Orion, Atorvastatin Parke-davis, Atorvastatin Ranbaxy 20mg, Atorvastatin Ranbaxy 20mg (atorvastatin) Unknown, Atorvastatin Sodium, Atorvastatin Tablet, Atorvastatin Tablets, Atorvastatin ^adenylchemie^, Atorvastatin(40 Mg Qd), Atorvastatin(atorvastatin), Atorvastatin(nr), Atorvastatin-calcium, Atorvastatin-calcium-hydrate (atorvastatin), Atorvastatin/01326101(atorvastatin), Atorvastatin/01326101/(atorvastatin), Atorvastatin,placebo (code Not Broken), Atorvastatina, Atorvastatina (atorvastatin Calcium), Atorvastatina (atorvastatine), Atorvastatina (tablets), Atorvastatine, Atorvastatine (atorvastatin), Atorvastatine (atorvastatin) (atorvastatin), Atorvastatine (tahor), Atorvastatinn Film-coated Tablet (pfizer), Atorvastatinum, Atorvastation (lipitor), Atorvastation Calcium, Atorvastation Calcium (atorvastatin Calcium), Atorvastation(atorvastatin), Atorvastatn, Atorvastatn Cacium, Atorvastatn Calcium, Atorvastatni, Atorvastin, Atorvastin Sodium, Atorvastina (atorvastatin), Atorvastine, Atorvastitine, Atorvasttain (atorvastatin Calcium), Atorvasttin, Atorvasttin (atorvastatin), Atorvasttin Calcium, Atorvasttin Calcium (atorvastatin Calcium), Atorvatastatin (atorvastatin), Atorvatatin /01326101/, Atorvatatin (atorvastatin), Atorvatatin (unknown), Atorvatatin Calcium, Atorvistatin, Atorvostatin, Atorvstatin (10mg), Atorvstatin (atorvastatin), Atorzastatin (nr), Atotvastatin, Atovastatin, Atovastatin (atorvastatin), Atovastatin (atovastatin), Atovastatin Calcium, Atovastatin Calcium) (atovastatin Calcium), Atovrastatin Calcium, Atprvastatin, Atqrvastat1n (atorvastatin), Atroastatin Calcium, Atrorvastatina (atorvastatin Calcium), Atrovastatin, Atrovastatin (atorvastatin Calcium), Atrovastatin (atorvastatin), Atrovastatin (atrovastatin), Atrovastatin (atrovastatin) (atrovastatin), Atrovastatin Caclium, Atrovastatin Calcium, Atrovastatin Calcium (atorvastatin Calcium), Atrovastatin Calcium (lipitor), Atrovastatin Calcium Hydrate, Atrovastatin Unk, Atrovastatin(atorvastatin), Atrovastatine, Atrovastin, Atrovastine, Atrvastatine (atrovastatin Calcium), Blinded Atorvastatin Calcium, Calcic Atorvastatine, Calcium/lipitor, Cardyl, Cardyl (atorvastatin), Cardyl (atorvastatin Calcium) (atorvastatin Calcium), Cardyl (atorvastatin Calcium) Tablet, 40 Mg, Cardyl (atorvastatin Calcium)(10 Milligram, Tablets)(atorvastatin Calc, Cardyl (atorvastatin), Cardyl (atorvastatin) Film-coated 20mg Tablet, Cardyl (cardyl), Cardyl (con.), Cardyl (tablets), Cardyl Comprimido Recubiertos Con Pelicula (atorvastatin Calcium Trihy, Cardyl/ Atorvastatine, Citalor, Citalor (atorvastatin), Citalor (atorvastatin), Citalor (atorvastatin), Citalor (atorvastatin), Citalor (atorvastatin), Citalor (atorvastatin), Citalor 9atorvastatin), Citalor(atorvastatin), Clean Study-cetp Inhibitor/atorvastatin/ Placebo-, Co Atorvastatin, Cotalor (atorvastatin), Diaatorvastatina, Generic Lipitor, Generic Name For Lipitor, L,ipitor (atorvatatin), Libitor, Liipitor (atorvastatin Calcium), Liitor (atorvastatin), Lipator 200 Mg, Lipator () Atorvastatin, Lipator (atorvastatin Calcium), Lipator (atorvastatin), Lipator (atorvastatin) Tablets, Lipator 200 Mg, Lipidor, Lipiitor (atorvastatin Calcium), Lipior (atorvastatin Calcium), Lipior (atorvastatin), Lipiotor /net/ (atorvastatin Calcium), Lipiotr, Lipirot (atorvastatin), Lipitcr (atorvastatin Calcium, Lipitior, Lipitior (atorvastatin Calcium), Lipititor (atorvastatin), Lipitlor, Lipito (atorvastatin), Lipito(atorvastatin), Lipitoe (atorvastatin Calcium), Lipitoir (atorvastatin Calcium), Lipitol, Lipitol (atorvastatin Calcium), Lipiton (atorvastatin), Lipitopr (atorvastatin Calcium), Lipitor, Lipitor /net/, Lipitor /net/, Lipitor /01326101/, Lipitor /01326102/, Lipitor (atorvastatin Calcium) (20 Milligram, Lipitor (atorbastatin), Lipitor /net/, Lipitor /01326101/ (atorvastatin), Lipitor (atorvastatin) Unspecified, Lipitor /net/(atorvastatin Calcium), Lipitor (atorvastatin), Lipitor (atorvastatin) (10 Milligram), Lipitor /net/ (atorvastatin Calcium), Lipitor /01326101/(atorvastatin), Lipitor /net/ (atorvastatin Calcium) Tablet, Lipitor /net/, Lipitor /net/ (atorvastatin Calcium), Lipitor /net/(atorvastatin Calcium), Lipitor / Net/(atorvastatin Calcium), Lipitor /01326101/, Lipitor (atrovastatin /01236101), Lipitor (torvastatin, Lipitor / Net/ (atorvastatin Calcium), Lipitor /01326101/ (atorvastatin), Lipitor /net/, Lipitor /net/ (atorvastatin Calcium), Lipitor ( To Unknown), Lipitor (10 Milligram), Lipitor (10 Milligram, Tablets), Lipitor (20 Milligram), Lipitor (20 Milligram, Tablets), Lipitor (80 Milligram), Lipitor (aorvastain), Lipitor (aorvastatin), Lipitor (aotravastatin), Lipitor (aotravatatin), Lipitor (aotrvastatin Calcium), Lipitor (aotrvastatin), Lipitor (aotrvastin Calcium), Lipitor (arorvastatin), Lipitor (artorvastatin Calcium), Lipitor (artorvastatin), Lipitor (atiorvastatin), Lipitor (atoevastatin Calcium Hydrate), Lipitor (atorastatin Calcium), Lipitor (atoravastatin Calcium), Lipitor (atoravstatin), Lipitor (atorcastatin), Lipitor (atorgastatin), Lipitor (atorvasatatin), Lipitor (atorvasatin Calcium), Lipitor (atorvastaatin), Lipitor (atorvastain Calcium), Lipitor (atorvastatatin Calcium), Lipitor (atorvastati Calcium), Lipitor (atorvastatin Calcium) (atorvastatin Calcium), Lipitor (atorvastatin /0132610), Lipitor (atorvastatin /01326101/), Lipitor (atorvastatin Ca,cium), Lipitor (atorvastatin Caclium), Lipitor (atorvastatin Caclium) Tablet, Lipitor (atorvastatin Calciu) Tablet, Lipitor (atorvastatin Calciuim), Lipitor (atorvastatin Calciuim) (atorvastatin Calcium), Lipitor (atorvastatin Calcium Hydrate), Lipitor (atorvastatin Calcium), Lipitor (atorvastatin Calcium) (20 Milligram) (atorvastatin Calcium), Lipitor (atorvastatin Calcium) (40 Milligram)(atorvastatin Calcium), Lipitor (atorvastatin Calcium) (atoravastatin Calcium), Lipitor (atorvastatin Calcium) (atorvastatin Calcium), Lipitor (atorvastatin Calcium) (atorvastin Calcium), Lipitor (atorvastatin Calcium) (atrovastatin Calcium), Lipitor (atorvastatin Calcium) (tablets) (atorvastatin Calcium), Lipitor (atorvastatin Calcium) Formulation Unknown, Lipitor (atorvastatin Calcium) Per Oral Nos, Lipitor (atorvastatin Calcium) Tablet, 10 Mg, Lipitor (atorvastatin Calcium) Tablet, Unknown, Lipitor (atorvastatin Calcium) Unk, Lipitor (atorvastatin Calcium) Unknown, Lipitor (atorvastatin Calcium) Unspecified, Lipitor (atorvastatin Calcium0, Lipitor (atorvastatin Calicum), Lipitor (atorvastatin Calicum) (atorvastatin Calcium), Lipitor (atorvastatin Calium), Lipitor (atorvastatin Claicum), Lipitor (atorvastatin Hydrochloride), Lipitor (atorvastatin Sodium) Tablet, Lipitor (atorvastatin), Lipitor (atorvastatin) ( 10 Milligram), Lipitor (atorvastatin) (10 Milligram), Lipitor (atorvastatin) (20 Milligram), Lipitor (atorvastatin) (40 Milligram) (atorvastatin), Lipitor (atorvastatin) (atorvastatin Calcium), Lipitor (atorvastatin) (nr), Lipitor (atorvastatin) (tablet) (atorvastatin), Lipitor (atorvastatin) (tablets) (atorvastatin), Lipitor (atorvastatin) (tam), Lipitor (atorvastatin) Per Oral Nos, Lipitor (atorvastatin) Tablet, Lipitor (atorvastatin) Unspecified, Lipitor (atorvastatin)(10 Milligrams), Lipitor (atorvastatin)q, Lipitor (atorvastatin0, Lipitor (atorvastation Calcium), Lipitor (atorvastatn Calcium), Lipitor (atorvastatni Calcium), Lipitor (atorvastatni), Lipitor (atorvastatom Calcium), Lipitor (atorvastator)), Lipitor (atorvasttin Calcium), Lipitor (atorvatatin Calcium), Lipitor (atorvatatin Clacium), Lipitor (atorvatatin), Lipitor (atotvastatin), Lipitor (atovastatin Calcium), Lipitor (atovasttin Calcium), Lipitor (atovrastatin Calcium), Lipitor (atrovasatin Calcium) (atrovastatin Calcium), Lipitor (atrovastatin /01326101/), Lipitor (atrovastatin Calcium) Tablet, Lipitor (atrovastatin Calcium) Tablet, 40 Mg, Lipitor (atrovastatin) Tablets, Lipitor (atrvastatin), Lipitor (con), Lipitor (con.), Lipitor (contin), Lipitor (lipitor), Lipitor (long Term), Lipitor (lopid), Lipitor (net/ (atorvastatin Calcium), Lipitor (prev.), Lipitor (storvastatin), Lipitor (unknown Until Continuing), Lipitor (unknown) Atorvastatin, Lipitor (unspecified) Atorvastatin, Lipitor - Atorvastatine, Lipitor - Pfizer Parke Davis, Lipitor -atorvastatine, Lipitor -generic-, Lipitor .net/(atorvastatin Calcium), Lipitor / Net/ (atorvastatin Calcium), Lipitor /00034001/, Lipitor /01326101 (atorvastatin), Lipitor /01326101/, Lipitor /01326101/ (atorvastatin), Lipitor /01326101/(atorvastatin), Lipitor /01326101/. Mfr: Not Specified, Lipitor /01326101/. Mfr: Not Specified, Lipitor /013261010/(atorvastatin), Lipitor /gb/, Lipitor /net (atorvastatin Calcium), Lipitor /net/, Lipitor /net/ (atorvastatin Calcium), Lipitor /net/ (atorvastatin Calcium), Lipitor /net/ (atorvastatin Calcium) Lasix (furosemide), Lipitor /net/ (atorvastatin), Lipitor /net/ (atorvastin Calcium), Lipitor /net/ (atovastatin Calcium), Lipitor /net/ (atrovastatin Calcium), Lipitor /net/(atorvastatin Calcium), Lipitor /unk/ (atorvastatin), Lipitor /unk/ (atorvastatin) Coated Tablet, Lipitor /unk/ (atorvastatin) Tablet, Lipitor /unk/ (atorvastatin0, Lipitor /unk/(atorvastatin), Lipitor 10 Mg Once Daily By Mouth, Lipitor 80 Miligrams Pfizer/caremark, Lipitor 9atorvastatin Calcium), Lipitor 9atorvastatin), Lipitor 9atorvastatin0, Lipitor Atorvastatin Capcium), Lipitor Atorvastatine, Lipitor Combo Vs Placebo, Lipitor Formulation Unknown, Lipitor Generic, Lipitor Mfr: Parke, Davis And Company, Lipitor Orifarm, Lipitor Tab 40 Mg(tablets), Lipitor Tablet Coated 20 Mg, Lipitor Therapy, Lipitor Tnt(atorvastatin), Lipitor Vs Placebo Study Drug, Lipitor(atorvastain Calcium), Lipitor(atorvastatin Calcium)( 20 Milligram) (atorvastatin Calcium), Lipitor(atorvastatine), Lipitor(atrovstatin Calcium), Lipitor. Mfr: Parke, Davis And Company, Lipitor/ Net/ (atorvastatin Calcium), Lipitor/0132610, Lipitor/01326101(atorvastatin0, Lipitor/01326101/ (atorvastatin), Lipitor/01326101/(atorvastatin), Lipitor/01326101/(atorvastin), Lipitor/gaudet' Contains Both 20 Mg, Lipitor/net (atorvastatin Calcium), Lipitor/net/ (atorvastatin Calcium), Lipitor/net/(atorvastatin Calcium), Lipitor/placebo (atorvastatin), Lipitor/unk/(atorvastatin), Lipitorq, Lipitors (atorvastatin), Lipitotr, Lipitpor (atorvastatin), Lipitpr (atorvastatin), Lipitro /01326101/ (atorvastatin), Lipittor (atorvastatin Calcium), Lipittor (atorvastatin), Liponorm (atorvastatin Calcium), Lipotor (atorvastatin), Lipox, Lipox Retard, Liprimar, Liprimar (tablets), Liptior, Liptior (atorvastatin Calcium), Liptiro (atorvastatin Calcium) (atorvastatin Calcium), Liptor (atorvastatin Calcium), Liptor (atorvastatin/01326101/), Liptor - Atorvastatin - Tablet - 10 Mg, Lipztor (atorvastatin), Lisipitor, Lispitor, Litorba (atorvastatin), Lkipitor (atorvastatin), Llipitor, Llipitor (atorvastatin), Lowden, Lowden (all Other Therapeutic Products), Oral Lipitor, Orvastatin Calcium (lipitor), Pfizer Lipitor, Placebo/atorvastatin, Prevencor, Prevencor /01326102/, Prevencor ( Atorvastatin Calcium ) (not Specified), Prevencor (atorvastatin Calcium), Prevencor (atorvastatin Calcium), Prevencor /01326102/, Prevencor (atorvastatin Calcium), Prevencor (atorvastatin Hydrochloride) (atorvastatin Calcium), Prevencor (atorvastatin), Prevencor (tablets), Prevencor /01326102/, Prevencor /01326102/ (atorvastatin Calcium), Prevencor /01326102/ (prevencor Atorvastatijn Calcium) (not Specified), Ratio-atorvastatin, Sortis, Sortis /01326101/, Sortis /01326102/, Sortis (atorvastatin), Sortis (atorvastatin), Sortis (atorvastin Calcium), Sortis (atorvastatin), Sortis (atorvastatin), Sortis ^goedecke^ (atorvastatin Calcium), Sortis (atorvasatin Calcium), Sortis (atorvastatin), Sortis (atorvastatin), Sortis (atorvastatin), Sortis Goedecke (atorvastatin Calcium), Sortis (atorvastatin), Sortis (film-coated ) (atorvastatin Calcium, Sortis (10 Milligram), Sortis (atorbastatin), Sortis (atorovastatin Calcium)(atorovastatin Calcium)(atorovastatin Ca, Sortis (atorvasatin Calcium), Sortis (atorvastatin Calcium), Sortis (atorvastatin Calcium) (10 Milligram, Tablet) (atorvastatin Cal, Sortis (atorvastatin Calcium) (10 Milligram, Tablets), Sortis (atorvastatin Calcium) (atorovastatin Calcium), Sortis (atorvastatin Calcium) (atrovastatin Calcium), Sortis (atorvastatin Calcium) (tablets), Sortis (atorvastatin Calcium) Film-coated Tablet, Sortis (atorvastatin Calcium) Tablets, Sortis (atorvastatin Calcium, , 0), Sortis (atorvastatin Sodium), Sortis (atorvastatin), Sortis (atorvastatin) (atorvastatin), Sortis (atorvastatin) (tablets) (atorvastatin), Sortis (atorvastatin) (tablets0 (atorvastatin), Sortis (atorvastatin0, Sortis (atovastatin) (atorvastatin), Sortis (atrovastatin), Sortis (con.), Sortis (nr), Sortis (sortis-atorvastatin Calcium) (not Specified), Sortis (tablet) (atorvastatin), Sortis (taf), Sortis / 01326101/ (sortis Atorvastatin Calcium) (not Specified), Sortis /01326101/, Sortis /01326101/ (atorvastatin), Sortis /01326102/, Sortis /01326102/ (sortis) (not Specified), Sortis 10, Sortis 10 (liporeduct), Sortis 10 (ta), Sortis 20, Sortis 20 (atorvastatin Calcium), Sortis 20 / Atorvastatin, Sortis 20 Mg, Sortis 40, Sortis 80 Mg, Sortis Goedecke, Sortis Tablets, Sortis ^goedecke^, Sortis ^goedecke^ (atorvastatin Calcium), Sortis ^goedecke^ (atorvastatin Calcium) Tablet, Sortis ^goedecke^ (atorvastatin Calcium, ), Sortis ^goedecke^ (atorvastatin Sodium), Sortis ^goedecke^ (atorvastatin), Sortis ^goedecke^ (atovarstatin Calcium), Sortis ^goedecke^ Atorvastatin Calcium), Sortis ^goedeck^ (atorvastatin Calcium), Sortis ^parke Davis^, Sortis ^parke-davis^, Sortis ^parke-davis^ (atorvastatin Calcium) Tablet, Sortis ^parke-davis^ (atorvastatin Calcium) Tablet, 10 Mg, Sortis ^parke-davis^ (avorvastatin Calcium), Sortis(atorvastatin Calcium), Sortis(atorvastatin Calcium) (10 Milligram, Tablet) (atorvastatin Calc, Sortis9ta), Statin (?lipitor/atorvastatin), Substitute For Lipitor, Tahor, Tahor (atorvastatin), Tahor (atorvastatin), Tahor (atorvastatin), Tahor (atorvastatin,), Tahor (atorvastatin), Tahor (atorvastatin), Tahor (atorvastatin), Tahor (atorvastatin), Tahor (40 Mg, Tablet) (atorvastatin Calcium), Tahor (atorvastatin), Tahor (atorvastatin), Tahor (atorvastatin), Tahor (atorvastatin), Tahor (atorvastatin), Tahor (atorvastatin), Tahor (atorvastatin), Tahor (atorvastatin Calcium) Unknown, Tahor (atorvastatin), Tahor (10 Mg, Film-coated Tablet) (atorvastatin Calcium), Tahor (10 Mg, Tablet) (atorvastatin), Tahor (10 Milligram, Tablets), Tahor (20 Mg, Coated Tablet) (atorvastin Calcium), Tahor (20 Mg, Tablet) (atorvastatin Calcium), Tahor (20 Mg, Tablet) (atorvastatin), Tahor (20 Milligram), Tahor (40 Mg, Coated Tablet) (atorvastatin Calcium), Tahor (40 Milligram, Tablets), Tahor (atoravastatin Calcium) (10 Milligram), Tahor (atorvastain), Tahor (atorvastatin , ), Tahor (atorvastatin Calciuim), Tahor (atorvastatin Calcium) (10 Mg, Tablet) (atorvastatin), Tahor (atorvastatin Calcium) (10 Milligram Tablets) (atorvastatin Calc, Tahor (atorvastatin Calcium) (10 Milligram), Tahor (atorvastatin Calcium) (40 Mg, Coated Tablet) (atorvastatin Calc, Tahor (atorvastatin Calcium) (40 Milligram), Tahor (atorvastatin Calcium) (atorvastatin Calcium), Tahor (atorvastatin Calcium) (atorvastatin), Tahor (atorvastatin Calcium) (atrovastatin Calcium), Tahor (atorvastatin Calcium) (nr), Tahor (atorvastatin Calcium) (tablet) (atorvastatin Calcium), Tahor (atorvastatin Calcium) (tablets) (atorvastatin Calcium), Tahor (atorvastatin Calcium) 10 Mg, Tahor (atorvastatin Calcium) 1u, Tahor (atorvastatin Calcium)(10 Milligram)(atorvastatin Calcium), Tahor (atorvastatin Calciuma) (atorvastatin Calcium), Tahor (atorvastatin Calicum), Tahor (atorvastatin Sodium), Tahor (atorvastatin), Tahor (atorvastatin) (10 Milligram (atorvastatin Calcium), Tahor (atorvastatin) (atorvastatin), Tahor (atorvastatin, , O), Tahor (atorvastatin, ), Tahor (atorvastatin, , 0), Tahor (atorvastatin, 0), Tahor (atorvastatin,), Tahor (atorvastatine), Tahor (atorvastation, ), Tahor (atorvastattin Acl, Tahor (atorvstatin), Tahor (atovastatin), Tahor (con.), Tahor (tablets), Tahor (tablets) (atorvastatin Calcium), Tahor (tahor - Atorvastatin Calcium), Tahor (tahor), Tahor (tahor-atorvastatin Calcium) (not Specified), Tahor - (atorvastatin Calcium) - Tablet - Unit Dose: Unknown, Tahor 10, Tahor 10 (atorvastatin Calcium), Tahor 40, Tahor 40 (atorvastatin Calcium), Tahor 40 Mg, Tahor 9atorvastatin Calcium) (10 Milligram, Tablet) (atorvastatin Calc, Tahor Film-coated Tablet, Tahor Film-coated Tablet 9atorvastatin), Tahor(atorvastatin), Tahor. Mfr: Not Specified, Tahor: Unknown / Atorvastatin, Teva-atorvastatin, Thahor (atorvastatin), Thaor (atorvastatin Calcium), Thor (atorvastatin) (atorvastatin), Torvacard, Torvacard (atorvastatin), Torvacard 20, Torvacard 40mg, Torvast, Torvast (atorvastatin), Torvast (20 Mg) (atorvastatin Calcium), Torvast (20 Mg)(atorvastatin Calcium), Torvast (atorvastatin Calcium), Torvast (atorvastatin Calcium) (atorvastatin Calcium), Torvast (atorvastatin Sodium), Torvast (atorvastatin), Torvast (atorvastatine), Torvast (atorvastatin_), Torvast (atrovastatin Calcium), Torvast(atorvastatin Calcium), Torvastatin, Torvastatina, Totalip, Totalip (atorvastatin Calcium), Totalip (atorvastatin), Totalip 40 Mg (atorvastatin Calcium), Totalip 40 Mg(atorvastatin Calcium), Totalip/atorvastative (), Ttorvastatin, Tulip, Tulip /00435301/, Tulip /01326102/, Tulip (atorvastatin), Tulip /00435301/, Zarator, Zarator /01326101/, Zarator (atorvastatin Calcium), Zarator (atorvastatin), Zarator (atorvastatin) (atorvastatin), Zarator (atorvastatin) Unknown, 80mg, Zarator (atrovastatin), Zarator (pfizer), Zarator /01326101/, Zarator 10, Zarator Film-coated Tablet, Zarator Film-coated Tablet (pfizer), Zarator ^parke Davis^, Zarator ^parke-davis^ (atorvastatin Calcium), Zarator ^pfizer^, Zarator ^pfizer^ (atorvastatin), Zarator ^pfizer^ (atorvastatin) Film-coated Tablet, Zorator

Lovastatin

FDA Listed Names (143):

(lovastatin Ratiopharm), ,ovastatin (lovastatin), Alticor, Altocor, Altocor 40mg Andrx, Altocor Andrx, Altocor (lovastatin) Extended-release (tablets), Altocor 20mg Andrx Labo, Altocor 40 Mg Andrx, Altocor 40mg Andrx, Altocor Andrx, Altocor Extended Release Tabs (lovastatin) Distributed By Andrx Labs I, Altoprev, Altoprev -lovastatin-, Altoprev (all Other Therapeutic Products) (60 Milligram), Altoprev (lovastatin), Altoprev 60, Altoprev Extended Release (watson Laboratories), Altoprev(lovastatin), Altoprev-er, Avostatin (lovastatin), Cardiostatin (lovastatin), Cardiostation, Lovastatin, Daltocor, Gen Lovastatin (lovastatin), Injectable Lovastatin + Serum, Lavastatin (lovastatin), Levastatin, Levastatin (lovastatin), Levistatin, Levostatin, Levostatin () Lovastatin, Levostatin (lovastatin), Levostatin (lovastatin) Unspecified, Levostatin 20 Mg, Lipivas (lovastatin), Liposcler (lovastatin), Lisinopril (lovastatin), Lochol (lovastatin), Lochol (lovastatin) Per Oral Nos, Louvastatin, Lova (lovastatin), Lovabeta (ngx) (lovastatin) Tablet, Lovabeta (ngx) (lovastatin) Tablet, 40mg, Lovacol (lovastatin), Lovasatatin, Lovasatatin (lovasatatin) (lovastatin), Lovasatin, Lovastaatin (lovastatin), Lovastain, Lovastan, Lovastan (lovastatin), Lovastat (lovastatin), Lovastatin, Lovastatin 40 Mg Daily Eon Labs, Lovastatin Lisinopril Lovastatin, Lovastatin (10 Milligram), Lovastatin (40 Milligram), Lovastatin (altocor Usa/), Lovastatin (con.), Lovastatin (formulation Unknown) (lovastatin), Lovastatin (lovasatin), Lovastatin (lovastain), Lovastatin (lovastatin) (10 Milligram, Tablets), Lovastatin (lovastatin) (20 Milligram,tablet) (lovastatin), Lovastatin (lovastatin) (40 Milligram), Lovastatin (lovastatin) (40 Milligrma), Lovastatin (lovastatin) (lovastatin), Lovastatin (lovastatin) (tablets), Lovastatin (lovastatin) (tablets) (lovastatin), Lovastatin (lovastatin) (unknown), Lovastatin (lovastatin) Tablet, Lovastatin (lovastatin) Unknown, Lovastatin (lovastatin)(lovastatin), Lovastatin (lovastatine), Lovastatin (lovastin), Lovastatin (lovstatin), Lovastatin (maternal Use), Lovastatin (unspecified) Lovastatin, Lovastatin 10 Mg Merck, Lovastatin 10 Mg, 20mg + 40 Mg Tablets, Lovastatin 20 Mg (blue), Lovastatin 20 Mg Daily, Lovastatin 40 Mg Purepac, Lovastatin 40mg Tab (sandoz), Lovastatin Actavis, Lovastatin By Lupin, Lovastatin Ct, Lovastatin Hctz, Lovastatin Tab, Lovastatin Tablets, Lovastatin(lovastatin) (80 Milligram), Lovastatin(lovastatin) Tablet, 20mg, Lovastatin-ht, Lovastatina, Lovastatina (lovastatin) (20 Milligram, Tablet) (lovastatin), Lovastatina ^cinfa^, Lovastatine, Lovastatinn (lovastatin), Lovastation, Lovastatni (lovastatin), Lovastatom, Lovastaton, Lovasterol, Lovastin, Lovastin (lovastatin), Lovastin (lovastin), Lovastine, Lovasttin (lovastatin), Lovaswtatin (lovastatin), Lovatat (lovastatin), Lovatatin, Lovatex (lovastatin), Lovatex (lovastatin), Lovestain, Lovestatin, Lovestatin (lovastatin) (lovastatin), Lovistatin, Lovistatin 2004 Stopped 8/10/04, Lovostatin, Lovostatin (40 Mg), Lovostatin (lovastatin), Lovstatin (lovastatin), Lvostatin, Mavecor, Mebacor, Mevaco (lovastatin), Mevaco(lovastatin), Mevacol, Mevacon (lovastatin), Mevacor, Mevacor (louvastatin), Mevacor (lovasatin), Mevacor (lovustatin), Mevacore, Mevastin (lovastatin), Mevcor, Mevicor, Mevlor (lovastatin), Mevocor, Mivacor, Use Of Unspecified Brand Lovastatin Since --/--/1995, ^levostatin^ Nos

Pravastatin

FDA Listed Names (534):

Alisor (pravastatin Sodium), Alsetin, Alsetin (pravastatin Sodium), Alsetin (pravastatin Sodium) Tablet, Alsetin (pravastatin Sodium) Tablet, 10 Mg, Alsetrin (pravastatin Sodium), Apo-pravastatin, Apo-pravastatine, Blisor (pravastatin Sodium), Bristacol (pravastatin Sodium), Cholstat, Cholstat /01341301/, Elisor, Elisor ( Pravastatin Soduim), Elisor (20 Mg) (pravastin Sodium), Elisor (40 Mg, Tablet) (pravastatin Sodium), Elisor (elisor - Pravastatin Sodium), Elisor (elisor - Pravastation Sodium) 20 Mg (not Specified), Elisor (pravasatin Sodium), Elisor (pravastatin Sodium) (20 Mg) (pravastatin Sodium), Elisor (pravastatin Sodium) (40 Mg, Tablet) (pravastatin Sodium), Elisor (pravastatin Sodium) (ta), Elisor (pravastatin Sodium) (tablets), Elisor (pravastatin Sodium) (tablets) (pravastatin Sodium), Elisor (pravastatin Sodium) (unk), Elisor (pravastatin Sodium) 20mg, Elisor (pravastatin Sodium) Ca, Elisor (pravastatin Sodium) Capsule, Elisor (pravastatin Sodium, , 0), Elisor (pravastatin Soidum), Elisor (pravstatin Sodium), Elisor (tablet) (pravastatin Sodium), Elisor (tablets) (pravastatin Sodium), Elisor Tabs, Elisor Tabs 20 Mg, Elisor(pravastatin Sodium) 20mg, Elisor- (pravastatin Sodium)- Tablet- 40 Mg, Generic Pravachol, Generic Prevachol, Ic Prevastatin 10 Mg, Apotex Usa , Inc., Ic Prevastatin 20 Mg, Apotex Usa , Inc., Lin-pravastatin Tabs, Lipemol, Lipemol (pravastatin Sodium), Lipemol Tabs, Lipemol Tabs 20 Mg, Liplat, Liplat (pravastatin Sodium), Liplat (pravastatin Sodium), Liplat (pravastatin Sodium) (pravastatin Sodium), Liplat (pravastin Sodium), Liplat Tabs, Liplat Tabs 10 Mg, Liplat Tabs 20 Mg, Lipostat, Lipostat (pravastatin, Lipostat (pravastatin Sodium), Lipostat (pravastatin Sodium) (pravastatin Sodium), Lipostat (pravastatin), Lipostat 10%, Lipostat Tabs, Lipostat Tabs 10 Mg, Maibastan, Maibastan (maibastan) (not Specified), Maibastan (pravastatin Sodium), Maibastan (pravastatin Sodium) (pravastatin Sodium), Maibastin, Maibbastan (pravastatin Sodium), Maibestan, Maxudin, Mebalotin (pravastatin Sodium), Mebalotin (con.), Mebalotin (pravastatin Sodium) (pravastatin Sodium), Mebalotin (pravastatin Sodium) Tablet, Melavotin, Melvalotin, Melvalotin (pravastatin Sodium), Mevalon (pravastatin Sodium), Mevalotin, Mevalotin /jpn/, Mevalotin (mevalotin- Pravastatin Na), Mevalotin /jpn/(parvastatin Sodium), Mevalotin (con.), Mevalotin (mevalotin), Mevalotin (mevalotin) (not Specified), Mevalotin (mevalotin) Tablet, Mevalotin (mevalotin) Tablet, 5 Mg, Mevalotin (mevalotin- Pravastatin Na), Mevalotin (pravastatin Sodiuim), Mevalotin (pravastatin Sodium( (ta), Mevalotin (pravastatin Sodium), Mevalotin (pravastatin Sodium) (nr), Mevalotin (pravastatin Sodium) (pravastatin Sodium), Mevalotin (pravastatin Sodium) (preparation For Oral Use (nos)) (prava, Mevalotin (pravastatin Sodium) (ta), Mevalotin (pravastatin Sodium) (tablet) (pravastatin Sodium), Mevalotin (pravastatin Sodium) Formulation Unknown, Mevalotin (pravastatin Sodium) Per Oral Nos, Mevalotin (pravastatin Sodium) Preparation For Oral Use(nos)) (pravast, Mevalotin (pravastatin Sodium) Tablet, Mevalotin (pravastatin Sodium) Tablet, 10 Mg, Mevalotin (pravastatin Sodium) Tablets, Mevalotin (pravastatin Sodium)(pravastatin Sodium), Mevalotin (pravastatin Sodium0, Mevalotin (pravastatin Soduim), Mevalotin (pravastatin Soidum), Mevalotin (pravastatin), Mevalotin (pravastation Sodium), Mevalotin (pravastatn Sodium), Mevalotin (pravstatin Sodium) Tablet, Mevalotin (prazastatin Sodium), Mevalotin (prevastatin Sodium), Mevalotin (provastatin Sodium), Mevalotin (prvastatin Sodium), Mevalotin (ta), Mevalotin (travastatin Sodium), Mevalotin (unknown), Mevalotin /jpn/, Mevalotin /jpn/ (pravastatin Sodium), Mevalotin Ipravastatin Sodium), Mevalotin Jpn (pravastatin Sodium) Tablet, Mevalotin Jpn (pravastatin Sodium) Tablet, 5 Mg, Mevalotin Tabs, Mevalotin Tabs 10 Mg, Mevalotin Tabs 20 Mg, Mevalotin(pravastatin Sodium) Per Oral Nos, Mevalotin/ Pravastatin Socium, Mevalotine (pravastatin Sodium), Mevan (pravastatin Sodium) Tablet, 10 Mg, Mevaolotin, Mevarich, Mevarich (pravastatin Sodium), Mevarich (pravastatin Sodium), Mevarich (pravastatin Sodium) (pravastatin Sodium), Mevarich Kaken, Mevarotin (pravastatin Sodium), Mevatorte, Mevatorte (pravastatin Sodium), Mevolotin (pravastatin Sodium), Novo Pravastatin, Novo-pravastatin, Paravastatin, Paravastatin Sodium, Paravastin, Parvastatin, Parvastatin Sodium (alsetin), Pavachol, Pavachol (pravastatin Sodium), Pavacohl, Pavacol, Pavasin (pravastatin Sodium), Pavastatin, Poravastatin, Praastatin, Praastatin Sodium, Prabastatin, Prabastatin (pravastatin), Prabastatin Sodium, Prabvastatin Na Amel (pravastatin Sodium), Pramevan, Pramevan (pravastatin Sodium), Pramevan (pravastatin Sodium) Tablet, Prareduct, Prareduct (pravastatin Sodium), Prareduct (pravastatin Sodium) (40 Milligram) (pravastatin Sodium), Prareduct (pravastatin Sodium) (pravastatin Sodium), Prasterol, Prasterol Tabs, Prava /soa/ (pravastatin Sodium), Pravacal, Pravachnl (pravastatin Sodium), Pravacho(pravastatin Sodium), Pravachol, Pravachol (20 Milligram), Pravachol (40 Milligram), Pravachol (cerivastatin Sodium), Pravachol (cholesterol- And Triglyceride Reducers), Pravachol (con.), Pravachol (finasteride), Pravachol (pracastatin Sodium), Pravachol (pravastain Sodium), Pravachol (pravastatin Calcium), Pravachol (pravastatin Hydrochloride), Pravachol (pravastatin Sodiuim), Pravachol (pravastatin Sodium) (20 Milligram), Pravachol (pravastatin Sodium) (40 Milligram), Pravachol (pravastatin Sodium) (ta), Pravachol (pravastatin Sodium) Unknown, Pravachol (pravastatin Sodium) Unspecified, Pravachol (pravastatin Sodium)`, Pravachol (pravastatin Soduim), Pravachol (pravastatin Sodum), Pravachol (pravastatin Soidum), Pravachol (pravastation Sodium), Pravachol (pravastatni Sodium), Pravachol (pravastatom Sodium), Pravachol (pravastin Sodium), Pravachol (pravasttin Sodium), Pravachol (pravistatin Sodium), Pravachol (pravsatatin Sodium), Pravachol (prev.), Pravachol - Pravastatin Sodium - Tablet - 40 Mg, Pravachol 10 Qd, Pravachol Tabs Rm 10 Mg, Pravachol. Mfr: Bristol-myers Squibb, Pravachol/pravastatin Sodium 20 Mg Po Teva Usa, Pravacol (pracastatin Sodium), Pravacol (pravastain Sodium), Pravacol (pravastatin Sodum), Pravacol (pravastatin), Pravacol Tabs, Pravacol Tabs 10 Mg, Pravacol/statin, Pravadual (pravastatin Sodium), Pravadual (pravastatin /w Acetylsalicylic Acid), Pravadual (pravastatin, Acetylsalicylic Acid), Pravadual (pravastatin/acetylsalicylic Acid) (tablets), Pravadual (pravastatine, Acetylsalicylic Acid), Pravadual Tabs, Pravadual Tabs 81 Mg/40 Mg, Pravalon (pravastatin Sodium), Pravalon (pravastatin Sodium), Pravalotin, Pravalotin (pravastatin Sodium), Pravalotin (pravastatin Sodium) (tablets) (pravastatin Sodium), Pravalotin (pravastatin Sodium) (tablets) (pravastatin), Pravalotin (pravastatin Sodium) Tablet, Pravalotin / Pravastatin Sodium, Pravapeak (pravastatin Sodium), Pravaron (pravastatin Sodium) Tablet, Pravasatatin Sodium (pravachol), Pravasatin, Pravasatin Sodium (pravastatin Sodium), Pravasatine, Pravaselect, Pravasin, Pravasin (pravastatin Sodium) 1, Pravasin (pravastatin Sodium) Tablet, Pravasin Protect, Pravasin Protect (pravastatin Sodium), Pravasin Protect Tabs 20 Mg, Pravasin Tabs 20 Mg, Pravasinc, Pravasine, Pravasine (pravastatin), Pravastain, Pravastain (pravastatin), Pravastain 20mg, Pravastain Sodium, Pravastain Sodium (pravastatin Sodium), Pravastan, Pravastan (pravastatin Sodium), Pravastan (pravastatin Sodium) (tablet) (pravastatin Sodium), Pravastan (pravastatin Sodium) Tablet, Pravastan (pravastatin Sodium) Tablet, 10 Mg, Pravastan (pravastatni Sodium), Pravastastin, Pravastastin Sodium (pravastatin Sodium), Pravastat, Pravastatatin, Pravastatin, Pravastatin (mfr: Unknown), Pravastatin /00880402/, Pravastatin (pravastatin Sodium) Unknown, Pravastatin (pravastatin Sodium) Unknown, Pravastatin (pravastatin Na), Pravastatin (ravastatin Sodium) Unknown, Pravastatin (pravastatin Na), Pravastatin (pravastatin) Tablet, Pravastatin (pravastatin Sodium) Unknown, Pravastatin (pravastatin) (tablets), Pravastatin (20 Milligram), Pravastatin (200 Mg) (pravastatin), Pravastatin (con.), Pravastatin (mfr: Unknown), Pravastatin (ngx), Pravastatin (paravastatin), Pravastatin (pracastatin), Pravastatin (pravaastatin), Pravastatin (pravastantin), Pravastatin (pravastatin Sodium), Pravastatin (pravastatin Sodium) (tablet) (pravastatin Sodium), Pravastatin (pravastatin Sodium) (tablet) (pravastatin), Pravastatin (pravastatin Sodium) Unknown, Pravastatin (pravastatin), Pravastatin (pravastatin) (20 Milligram), Pravastatin (pravastatin) (20 Milligram) (pravastatin), Pravastatin (pravastatin) (40 Milligram) (pravastatin), Pravastatin (pravastatin) (40 Milligram, Tablet) (pravastatin), Pravastatin (pravastatin) (40 Milligram, Tablets) (pravastatin), Pravastatin (pravastatin) (tablet) (pravastatin), Pravastatin (pravastatin) (tablets), Pravastatin (pravastatin) Formulation Unknown, Pravastatin (pravastatin) Ongoing, Pravastatin (pravastatin) Tablet, Pravastatin (pravastatin) Unknown, Pravastatin (pravastatin), 10 Mg, Pravastatin (pravastatin0, Pravastatin (provachol), Pravastatin (tablets), Pravastatin (unknwon), Pravastatin ,tablets 40 Mg, Pravastatin -pravachol-, Pravastatin 20 Mg, Pravastatin 20mg, Pravastatin 20mg (teva), Pravastatin 30 Mg, Pravastatin 40, Pravastatin 40 Mg Tablets (pravastatin), Pravastatin 5mg, Pravastatin A (pravastatin Sodium) (tablet) (pravastatin Sodium), Pravastatin Arrow, Pravastatin Dura, Pravastatin Family (pravastatin) Tablet, 20 Mg, Pravastatin Hexal, Pravastatin Kcl, Pravastatin Mr, Pravastatin Na (pravastatin Sodium), Pravastatin Na Amel (pravastatin Sodium), Pravastatin Natrium, Pravastatin Ranbaxy 10mg Tabletter, Pravastatin Ratioph, Pravastatin Ratiopharm, Pravastatin Sandoz, Pravastatin Sandoz Tablet, 10mg, Pravastatin Sandoz Tablet, 10mg, Pravastatin Socium, Pravastatin Sodim (pravastatin Sodium), Pravastatin Sodique (pravastatin), Pravastatin Sodiuim (pravastatin Sodium), Pravastatin Sodium, Pravastatin Sodium (mfr: Unknown), Pravastatin Sodium (pravachol), Pravastatin Sodium (0ravastatin Sodium), Pravastatin Sodium (20mg), Pravastatin Sodium (alsetin), Pravastatin Sodium (mfr: Unknown), Pravastatin Sodium (pravachol), Pravastatin Sodium (pravasatatin Sodium), Pravastatin Sodium (pravastatin Sodium) (20 Milligram, Tablets), Pravastatin Sodium (pravastatin Sodium) (pravastatin Sodium), Pravastatin Sodium (pravastatin Sodium) Tablet, Pravastatin Sodium (pravastatin Sodium) Unknown, Pravastatin Sodium (pravastatin Sodium, ,0), Pravastatin Sodium (pravastatine), Pravastatin Sodium (pravatin), Pravastatin Sodium (provastatin Sodium), Pravastatin Sodium (provastatin Sodium) 08/31/2009 To Unk, Pravastatin Sodium (ravastatin Sodium), Pravastatin Sodium (watson Laboratories), Pravastatin Sodium 10 Mg Tablets, Pravastatin Sodium 20 Mg Tablets, Pravastatin Sodium 40 Mg Tablets, Pravastatin Sodium 40 Mg Tablets (pravastatin), Pravastatin Sodium 40mg Taapo Ukn, Pravastatin Sodium Tablet (pravastatin), Pravastatin Sodium Tablets, Pravastatin Sodium Tablets (pravastatin Sodium), Pravastatin Sodium(pravachol), Pravastatin Sodium, 20 Mg, (1 In 1 D), Pravastatin Stada, Pravastatin Tablets (mfr: Unknown), Pravastatin Tablets 40 Mg (mfr: Unknown), Pravastatin Teva, Pravastatin ^squibb^ (pravastatin), Pravastatin(pravastatin) Unknown, Pravastatin-natrium, Pravastatin-ratiopharm 20 Mg, Pravastatina, Pravastatina Sodica, Pravastatina Stada, Pravastatine /00880401/, Pravastatine (pravastatin), Pravastatine (pravastatin Sodium), Pravastatine (pravastatin Sodium) (pravastatin Sodium), Pravastatine (pravastatin), Pravastatine (pravastatin) (pravastatin), Pravastatine (pravastatine) (pravastatine), Pravastatine /0088040/ (unknown), Pravastatine /00880401/, Pravastatine /00880401/ (pravastatin), Pravastatine Almus, Pravastatine Arrow, Pravastatine Biogaran, Pravastatine Na, Pravastatine Natrium, Pravastatine Ranbaxy 20mg Comprime Secable, Pravastatine Sel De Na (pravastatin Sodium), Pravastatine Sodique, Pravastatine Sodium, Pravastatine Tabs, Pravastatine Winthrop, Pravastatine Zydus, Pravastatine ^squibb^, Pravastatinenatrium Pch, Pravastatinenatrium Rp, Pravastating, Pravastatinum Natricum, Pravastation, Pravastation Sodium, Pravastatn, Pravastatn Sodium (pravastatin Sodium), Pravastatni (pravastatin) (40 Milligram, Tablets) (pravastatin), Pravastatni (pravastatin) (tablets) (pravastatin), Pravastatom, Pravastatrin, Pravastin, Pravastin (pravastin), Pravastin Sodium (pravacol), Pravastine, Pravastine Na, Pravastine Sandoz, Pravaststine, Pravaststinenatrium, Pravasttatin Sodium, Pravasttin, Pravat (pravastatin), Pravatatin Sodium, Pravatin (pravastatin Sodium), Pravatin (pravastatin Sodium), Pravatin (pravastatin Sodium) (tablet) (pravastatin Sodium), Pravatin (pravastatin Sodium) (tablet)(pravastatin Sodium), Pravatin (pravastatin Sodium) Tablet, Pravatin (pravastatin), Pravatin Solution, Pravator, Pravator 40 Mg, Pravaxhol (pravastatin Sodium), Pravcahol (pravastatin Sodium), Pravchol (pravastatin Sodium), Pravistatan, Pravistatin, Pravistatin Sodium (pravastatin Sodium), Pravitin (pravastatin Sodium) (10 Milligram) (pravastatin Sodium), Pravochal, Pravocol, Pravocol (pravastatin), Pravsatatin, Pravstatin, Pravstatin (pravastatin Sodium), Pravstatin (pravastatin) (nr), Pravstatin Sodium, Pravstatin Sodium (pravastatin Sodium), Pravustatin, Prazastain, Prazastatin, Prazastatin Sodium, Prazaxstatin, Prestatin (pravastatin Sodium), Prestatin (pravastatin), Prevacal, Prevachol, Prevachol (pravastatin Sodium), Prevachol (pravastatin Sodium) (pravastatin Sodium), Prevacol, Prevastain, Prevastat, Prevastatin, Prevastatin (pravastatin), Prevastatin Na, Prevastatin Sodium, Prevastatin Sodium (pravachol), Prevastin, Prevechol, Prevocal, Prevocol, Prevstatin, Prilostatin (presumed Pravastatin), Probastatin, Provacal, Provachol, Provachol (cholesterol- And Triglyceride Reducers), Provachol (cholesterol- And Triglycerides Reducers), Provachol (pravastatin Sodium), Provacl, Provacol, Provastatin, Provastatin (pravastatin), Provastatin Dont Have With Me At The Moment Dont Have With Me At The M, Provastatin Sodium (pravastatin Sodium), Provastatin, 40 Mg Chlorlestorel, Provastatin, 40 Mg Chlorlestorel, Provastatin-20, Provastatin/provachol, Provastatine, Provastin, Provatatin Sodium (provastatin Sodium) (pravastatin Sodium), Provical, Provicol, Provocal, Provochal, Provostatin (pravastatin) (con.), Provostatine, Prvasatatine Na (pravastatin Na - Pravastatin Sodium) 10 Mg (not Speci, Prvastatin, Prvastatin (pravastatin), Prvastatin Sodium, Rpavachol (pravastatin Sodium), Selectin (pravastatin Sodium), Selektine, Selektine (pravastatin Sodium), Selektine (pravastatin Sodium), Selektine (pravastatin Dodium), Selektine (pravastatin Sodium), Selektine (pravastatin Sodium) Tablet, Selektine (tablets), Selektine Tablet 20mg, Selektine Tabs, Selipran, Selipran (prabastatin Sodium), Selipran (pravastatin Sodium), Selipran (pravastatin Sodium) (40 Milligram, Tablets), Selipran (pravastatin Sodium) Tablet, 40 Mg, Selipran 20 Mg Tabletten (pravastatin), Selipran Tabs, Selipran Tabs 20 Mg, Selpran 20 Mg (pravastatin Na) Pravastatin Sodium), Sodium Pravastatin, Tab Mevalotin (pravastatin Na), Tab Mevalotin 10 Mg, Tab Pravastatin, Tatsuplamin (pravastatin Sodium), Vastein (pravastatin Sodium), Vasten (pravastatin Sodium) (40 Milligram), Vasten (pravastatin Sodium) (prevastatin Sodium), Vasten (pravastatin Soduim), Vasten (pravastatin), Vasten (pravastatin) (20 Milligram, Tablets), Vasten (pravasttin Sodium), Vasten (tablet) (pravastatin Sodium), Vasten - (pravastatin Sodium) - Tablet - 20 Mg, Vasten /00880402/ (pravastatin Sodium), Vasten(pravasatin Sodium), Viasten (pravastatin Sodium)

Simvastatin

FDA Listed Names (784):

(simvastatin), (simvastatin) - Unknown - 20 Mg, (simvastatin-mepha), (simvastattn), (simvastin-mepha), *simvastatin, Adco-simvastatin, Adco-simvastatin (simvastatin), Apo-simva, Apo-simvastatin, Arrow-simva (mah: Arrow Pharmaceuticals (nz) Ltd.), Arrow-simva 40mg (mah: Arrow Pharmaceuticals (nz) Ltd.), Asimvastatin, Calcium (unspecified Simvastatin), Cardin (simvastatin), Cardin/pol (simvastatin), Co Simvastatin, Co-simvastatin, Colemin, Colemin (simvastatin), Doc-simvastata, Docsimvasta, Docsimvastatine, Gen-simvastatin, Generic For Zocar, Generic For Zocor, Generic Simvastatin, Generic Zocor, Hypolipidemia Drug (simvastatin), Ic Simvastatin 40 Mg Tablet Tev Don't See It, Imvastatin (simvastatin), Li Povas (simvastatin) (simvastatin), Liipovas (simvastatin), Lipcut, Lipcut (simvastatin) Tablet, 10mg, Lipcut (ngx), Lipex, Lipex /00848101/, Lipex (simvastatin ) (40 Mg), Lipex (simvastatin) (simvastatin), Lipex (simvastatin) (ta), Lipidex (simvastatin), Lipo-off (simvastatin), Lipo-off (simvastatin) (simvastatin), Lipo-off (simvastatin)(simvastatin), Lipodown (simvastatin), Liponorm (simvastatin), Lipora M (simvastatin) (simvastatin), Lipovaas (simvastatin), Lipovas, Lipovas /00499301/, Lipovas /00848101/, Lipovas Banyu, Lipovas Banyu (simvastatin), Lipovas Banyu (simvastatin Ratiopharm), Lipovas Banyu (simvastatin), Lipovas Banyu (simvastatin), Lipovas (being Queried), Lipovas (japan), Lipovas (simvastatin Ratiopharm), Lipovas (simvastatin), Lipovas (simvastatin) (simvastatin), Lipovas (simvastatin) (tablets), Lipovas (simvastatin) Tablet, Lipovas (simvatatin) (ta), Lipovas (smivastatin) Tablet, Lipovas (ta), Lipovas /00848101/, Lipovas /00848101/ (simvastatin), Lipovas Banyu (simvastatin), Lipovas Tablets, Lipovas ^banyu^, Lipovas ^banyu^ (simvastatin), Lipovas ^banyu^ (simvastatin) Tablet, Lipovas ^banyu^ (simvastin), Lipovas ^banyu^, (simvastatin), Lipovatol (simvastatin), Lipozart (simvastatin), Lochol (simvastatin) Tablet, Lodales, Lodales (simvastatin) (1 Dosage Forms, Tablets), Lodales (simvastatin) (simvastatin), Lodales (simvastatin) Tablet, 20mg, Lodales (simvastatin,, 0), Lodales (simvastatin0, Lodales - (simvastatin) - Tablet - 40 Mg, Lodales 20, Lodales 20 Mg, Lodales 40 (simvastatin), Lodales(film Coated Tablet, Simvastatin), Manipulated Simvastatin, Medipo, Medipo (simvastatin), Misvastatin, Novo-simvastatin, Novosimvastatin, Ramian, Ramian (simvastatin), Ranzolont Film Coated Tablet 10mg (simvastatin) Tablet, 10 Mg, Redusterol (simvastatin), Riva Simvastatin (simvastatin), Riva-simvastatin, Rivasimvastatin (simvastatin), Samvastin, Semivastatin 80 Mg, Siimvastatin, Simavastatin, Simavistatin, Simavstatin, Simavstatin (simvastatin), Simbastastin, Simbastatin, Simbastatin (simvastatin), Simbastatin(simvastatin) (simvastatin), Simbvahexal, Simbvastatin (simvastatin), Simcalmed (simvastatin), Simcalmed (simvastatin) (simvastatin), Simcora (simvastatin), Simcore (simvastatin), Simfastatin, Simivastatin, Simivastatin (simvastatin), Simivastin, Simovastatin, Simovil (simvastatin), Simovil (simvastatin), Simovil (simvastatin), Simovl (simvastatin), Simuvastatine, Simva, Simva (simvastatin), Simva (simvastatin) (simvastatin), Simva 40, Simva Basics, Simva Basics 10mg Filmtabletten, Simva Hennig, Simva Henning, Simva Tad, Simva-hennig, Simvaastatin, Simvabeta, Simvabeta (simvabeta - Simvastatin) (not Specified), Simvabeta (simvastatin), Simvabeta (unknown), Simvabeta 20 Mg, Simvabeta 20 Mg Filmtabletten (simvastatin), Simvabeta 30, Simvabta (simvastatin), Simvacard, Simvacard (simvastatin), Simvacard (simvastatin), Simvacard (simvastatin), Simvacol, Simvacop, Simvacor, Simvacor (simvastatin Ratiopharm), Simvacor (simvastatin), Simvacor(simvastatin), Simvacort, Simvador, Simvador (simvastatin), Simvador (tablets), Simvadura, Simvadura 290, Simvaetatin, Simvagamma, Simvagamma 40, Simvahexal, Simvahexal (simvastatin), Simvahexal (simvahexal - Simvastatin), Simvahexal (simvastatin) Film-coated Tablet, 20mg, Simvahexal (simvastatin), Simvahexal (simvastatin) Film-coated Tablet, Simvahexal (40mg), Simvahexal (con.), Simvahexal (ngx), Simvahexal (ngx) (simvastatin) Film-coated Tablet, 40mg, Simvahexal (ngx)(simvastatin) Film-coated Tablet, 10mg, Simvahexal (simvahexal - Simvastatin), Simvahexal (simvastatin), Simvahexal (simvastatin) (10 Milligram), Simvahexal (simvastatin) (simvastatin), Simvahexal (simvastatin) Film-coated Tablet, Simvahexal (taf), Simvahexal 20 Mg, Simvahexal 40, Simvahexal 40 Mg Filmtabletten, Simvahexal 40 Mg Filmtabletten (simvastatin), Simvahexal 40mg Filmatabletten, Simvahexal(simvastatin), Simvalip, Simvanstatin, Simvar, Simvasatatin, Simvasatatin 10mg Tablets (simvastatin) Unknown, Simvasatin, Simvasatin (simvastatin), Simvasatin (simvastatin) (simvastatin), Simvasatin(simvastatin), Simvascor, Simvasin, Simvasin (simvastatin), Simvasin / Simvastatin, Simvast (simvastatin) (simvastatin), Simvasta, Simvastad, Simvastad (simvastatin), Simvastad 40 Mg Filmtabletten, Simvastain, Simvastain (simvastatin), Simvastaitn (simvastatin), Simvastan, Simvastastatin, Simvastastin, Simvastastin (strengths Unknown), Simvastat, Simvastatain, Simvastaten, Simvastatiin, Simvastatiin (simvastatin), Simvastatiini Ennapharma, Simvastatim, Simvastatim (simvastatin), Simvastatin, Simvastatin Film Coated, Simvastatin (simvastatin) Uknown, Simvastatin (simvasttin), Simvastatin (simvastatin) (tablets), Simvastatin (simvastatin) Unknown, 20mg, Simvastatin (simvastatin), Simvastatin (simvastatin) (tablets), Simvastatin (mfr Unknown), Simvastatin Orifarm (simvastatin Ratiopharm), Simvastatin Orifarm (simvastatin Ratiopharm), Simvastatin 20mg Dr. Reddy's La, Simvastatin 'novo', Simvastatin ( To Continuing), Simvastatin (08/30/2011 To Continuing), Simvastatin (10 Mg, Tablets)(simvastatin), Simvastatin (10 Milligram), Simvastatin (20 Milligram), Simvastatin (40 Miligram), Simvastatin (40 Milligram), Simvastatin (40 Milligram, Tablets), Simvastatin (arrow), Simvastatin (aurobindo), Simvastatin (blinded), Simvastatin (con), Simvastatin (con.), Simvastatin (formulation Unknown), Simvastatin (isimvastatin) (simvastatin), Simvastatin (jnj16269110 Comparator) Tablet, Simvastatin (lipovas), Simvastatin (lodales), Simvastatin (mfr Unknown), Simvastatin (ngx), Simvastatin (ngx) (simvastatin) Film-coated Tablet, Simvastatin (ngx)(simvastatin) Tablet, 20mg, Simvastatin (no Label), Simvastatin (nr), Simvastatin (pill), Simvastatin (pms), Simvastatin (sanofi), Simvastatin (siimvastatin), Simvastatin (simastatin), Simvastatin (simavastatin), Simvastatin (simavastatin) (simvastatin), Simvastatin (simcastatin), Simvastatin (simivastatin), Simvastatin (simmastatin), Simvastatin (simvacor) (simvastatin), Simvastatin (simvasatin) (simvastatin), Simvastatin (simvasstatin), Simvastatin (simvastain), Simvastatin (simvastatin (simvastatin), Simvastatin (simvastatin (tablet) (simvastatin), Simvastatin (simvastatin Ratiopharm), Simvastatin (simvastatin), Simvastatin (simvastatin) (10 Milligram Tablet) (simvastatin), Simvastatin (simvastatin) (10 Milligram), Simvastatin (simvastatin) (10 Milligram) (simvastatin), Simvastatin (simvastatin) (10 Milligram, Tablet) (simvastatin), Simvastatin (simvastatin) (20 Millgiram) (simvastatin), Simvastatin (simvastatin) (20 Milligram), Simvastatin (simvastatin) (20 Milligram) (simvastatin), Simvastatin (simvastatin) (20 Milligram, Tablets) (simvastatin), Simvastatin (simvastatin) (40 Milligram), Simvastatin (simvastatin) (40 Milligram) (simvastatin), Simvastatin (simvastatin) (40 Milligram, Tablet) (simvastatin), Simvastatin (simvastatin) (40 Milligram, Tablets)(simvastatin), Simvastatin (simvastatin) (8 Milligram), Simvastatin (simvastatin) (80 Milligram) (simvastatin), Simvastatin (simvastatin) (80, Tablets), Simvastatin (simvastatin) (con.), Simvastatin (simvastatin) (nr), Simvastatin (simvastatin) (prev.), Simvastatin (simvastatin) (simvasatatin), Simvastatin (simvastatin) (simvasatin), Simvastatin (simvastatin) (simvastatin), Simvastatin (simvastatin) (suspension) (simvastatin), Simvastatin (simvastatin) (tablet) (simvastatin), Simvastatin (simvastatin) (tablets), Simvastatin (simvastatin) (tablets) (simvastatin), Simvastatin (simvastatin) (unknown), Simvastatin (simvastatin) 07nov2011:10feb2012, Simvastatin (simvastatin) 80mg, Simvastatin (simvastatin) Dose, Form, Route And Frequency Unknown: Ora, Simvastatin (simvastatin) Film Coated Tablet, 40 Mg, Simvastatin (simvastatin) Film Tablet, Simvastatin (simvastatin) Film-coated Tablet, Simvastatin (simvastatin) Ongoing, Simvastatin (simvastatin) Oral Solution, Simvastatin (simvastatin) Simvastatin (simvastatin), Simvastatin (simvastatin) Tablet, Simvastatin (simvastatin) Tablet, 10mg, Simvastatin (simvastatin) Tablet, 40 Mg, Simvastatin (simvastatin) Tablets, Simvastatin (simvastatin) Unknown, 20mg, Simvastatin (simvastatin) Unknown:unknown, Simvastatin (simvastatin) Unspecified, Simvastatin (simvastatin) [10057097 - Drug Use For Unknown Indication], Simvastatin (simvastatin)(20 Milligram)(simvastatin), Simvastatin (simvastatin)(tablets)(simvastatin), Simvastatin (simvastatin, , 0), Simvastatin (simvastatin, 40 Mg, Daily), Simvastatin (simvastatin, Simvastatin) (simvastatin, Simvastatin), Simvastatin (simvastatin0, Simvastatin (simvastatin0 (simvastatin), Simvastatin (simvastatins), Simvastatin (simvastatn), Simvastatin (simvastatni), Simvastatin (simvastattn), Simvastatin (simvastin), Simvastatin (simvasttin), Simvastatin (simvatatin), Simvastatin (sinvacor)(simvastatin), Simvastatin (sinvascor), Simvastatin (sinvastatin), Simvastatin (sivastatin), Simvastatin (smivastatin), Simvastatin (smvastatin), Simvastatin (smvastatin) (tablets), Simvastatin (somatropin), Simvastatin (statins), Simvastatin (stmvastatin), Simvastatin (strength Unknown), Simvastatin (unknown), Simvastatin (watson Laboratories), Simvastatin (zocor), Simvastatin (zocor) (simvastatin), Simvastatin (zocor) Tab, Simvastatin (zocor, 20 Mg), Simvastatin - Simcombin, Simvastatin - Unknown - 20 Mg, Simvastatin -zocor- 40 Mg Simvastatin 40mg Tab, Simvastatin /00885601/, Simvastatin 10, Simvastatin 10mg Medco, Simvastatin 10mg Tablets, Simvastatin 1a Farma (ngx), Simvastatin 1a Farma (ngx) (simvastatin) Film-coated Tablet, 40mg, Simvastatin 1a Pharma (ngx), Simvastatin 1a Pharma (ngx)(simvastatin) Film-coated Tablet, 40mg, Simvastatin 1a Pharma Gmbh (simvastatin), Simvastatin 20 Mg 1x Daily, Simvastatin 20 Mg American Health, Simvastatin 20 Mg Pill Box Drugs, Simvastatin 20 Mg Reddy, Simvastatin 20 Mg Zydus Pharma, Simvastatin 20 Mg. Daily Ranbaxy, Simvastatin 20 Mg. Generic For Zocor, Simvastatin 20mg 1 Qd, Simvastatin 20mg Don Know, Simvastatin 20mg Dr Reddy, Simvastatin 20mg Dr. Reddy's La, Simvastatin 20mg Tablet, Simvastatin 20mg Tablet Tev, Simvastatin 20mg Tablets, Simvastatin 20mg/daily, Simvastatin 35 Mg, Simvastatin 40, Simvastatin 40 (simvastatin), Simvastatin 40 Mg (simvastatin), Simvastatin 40 Mg Dr Reddy's Lab, Simvastatin 40 Mg Drl, Simvastatin 40 Mg Tab(simvastatin), Simvastatin 40 Mg Tabl Nn, Simvastatin 40 Mg Teva, Simvastatin 40 Mg Teva Usa St-dea Ar6353990, Simvastatin 400 Mg Tab, Simvastatin 40mg Nightly Oral, Simvastatin 40mg Tablets (simavastatin) Unknown, Simvastatin 40mg Teva, Simvastatin 40mg Walgreens, Simvastatin 5 Mg Tablets (simvastatin), Simvastatin 50 Mg, Simvastatin 5mg Tablets, Simvastatin 6/12/08 Meva, Simvastatin 80 Generic, Simvastatin 80 Mg Po Qhs (generic), Simvastatin 80 Mg Us Vet Adm Ndc#00093715698, Simvastatin 80 Teva Usa, Simvastatin 80mg 1 Po Qd, Simvastatin 80mg Tablets Teva, Simvastatin 920 Mg, 1/1day), Simvastatin 9simvastatin), Simvastatin 9simvastatn0, Simvastatin ??/??/2003, Simvastatin Aco, Simvastatin Aco (simvastatin), Simvastatin Actavis, Simvastatin Actavis (simvastatin), Simvastatin Actavis (simvastatin) (10 Milligram, Tablets) (simvastatin, Simvastatin Al, Simvastatin Al / Simvastatin, Simvastatin Alternova (simvastatin), Simvastatin Antilipemic Agents, Simvastatin Arrow, Simvastatin Aurobindo, Simvastatin Biogaran, Simvastatin Corax, 40 Mg, Filmtabl., Simvastatin Ct, Simvastatin Ct (simvastatin), Simvastatin Family, Simvastatin Family (simvastatin )tablet, 20 Mg, Simvastatin Family (simvastatin) Tablet, Simvastatin Family (simvastatin) Tablet, 10mg, Simvastatin Film Coated Tablet (simvastatin), Simvastatin Film Coated Tablet (simvastatin) Tablet, Simvastatin Film Coated Tablet 20mg (simvastatin), Simvastatin Film Coated Tablet 20mg (simvastatin) Tablet, 20 Mg, Simvastatin Film Coated Tablets, Simvastatin Film-coated (mfr: Unknown), Simvastatin Film-coated (mfr: Unknown), Simvastatin Film-coated Tablet (aurobindo), Simvastatin Genericon Pharma, Simvastatin Generics, Simvastatin Heumann, Simvastatin Heumann (tablet) (simvastatin), Simvastatin Hexal (simvastatin), Simvastatin Hexal (ngx) (simvastatin) Film-coated Tablet, 40mg, Simvastatin Hexal (simvastatin), Simvastatin I, Simvastatin Krka, Simvastatin Ksk (simvastatin), Simvastatin Lidex, Simvastatin Mepha, Simvastatin Merck And Co, Simvastatin Nobeta, Simvastatin Ns, Simvastatin Pch, Simvastatin Q-ph 40mg Fta, Simvastatin Ranbaxy 10mg Filmdragerad Tablett, Simvastatin Ranbaxy 20mg Filmtabletten, Simvastatin Ranbaxy 40mg, Simvastatin Ranbaxy Film Coated Tablets 10mg, Simvastatin Ratio, Simvastatin Rationpharm, Simvastatin Ratiopharm, Simvastatin Ratiopharm (simvastatin Ratiopharm), Simvastatin Ratiopharm (simvastatin), Simvastatin Rx 10 Mg 093, Simvastatin Rx 20 Mg 540, Simvastatin Rx 40 Mg 879, Simvastatin Rx 5 Mg 613, Simvastatin Rx 80 Mg 292, Simvastatin Sandoz, Simvastatin Sandoz (ngx), Simvastatin Sandoz (ngx) (simvastatin ) Film-coated Tablet, 20mg, Simvastatin Sandoz (ngx) (simvastatin), Simvastatin Stada, Simvastatin Tab, Simvastatin Tablet, Simvastatin Tablet (simvastatin), Simvastatin Tablet (simvastatin) Tablet, Simvastatin Tablet 10mg, Simvastatin Tablet 20 Mg Aurobindo, Simvastatin Tablet 40 Mg (simvastatin), Simvastatin Tablet 40 Mg (simvastatin) Tablet, Simvastatin Tablet 40mg (simvastatin) Tablet, Simvastatin Tablet 80mg, Simvastatin Tablets 5mg Meek, Simvastatin Tablets 80 Mg / Unknown, Simvastatin Tablets Usp 40 Mg (simvastatin Tablets 40mg) (40 Milligram, Simvastatin Tablets Usp, 20mg (atllc), Simvastatin Tablets Usp, 40mg (atllc) (simvastatin), Simvastatin Tablets, 20 Mg, Simvastatin Tabs 40mg Teva, Simvastatin Tabs 40mg Watson Labs, Simvastatin ^krka^, Simvastatin ^orifarm^ (simvastatin Ratiopharm), Simvastatin ^paranova^, Simvastatin ^wolff^ (simvastatin), Simvastatin(nos), Simvastatin(simvastatin) Unk To Unk, Simvastatin(simvastatin) (10 Milligram), Simvastatin(simvastatin) Tablet, Simvastatin(simvastatn), Simvastatin, 20 Mg, Tabl, Nn, Simvastatin, 40 Mg (mfr: Unknown), Simvastatin, Tabl, Nn, Simvastatin-generic-, Simvastatin-mepha, Simvastatin-mepha (simvastatin), Simvastatin-ranbaxy Jacksonville Fl 32257 Also Mfg By Ohmdah North Bru, Simvastatin-ratiopharm, Simvastatin-ratiopharm (simvastatin), Simvastatin,,,antilipemic Agents,tablets,,, Simvastatina, Simvastatina (simvastatin), Simvastatina Davur (simvastatin) (simvastatin), Simvastatina Mepha, Simvastatina Mepha (simvastatin), Simvastatina Normon, Simvastatina Ranbaxy 10mg Compresse Rivestite Con Film, Simvastatina Ranbaxy 20mg Comprimidos Efg, Simvastatina Ranbaxy 5mg Compresse Rivestite Con Film, Simvastatina Sandoz, Simvastatina(simvastatin), Simvastatine, Simvastatine (simvastatine), Simvastatine Ranbaxy 5mg (simvastatin) Tablet, 5 Mg, Simvastatine (all Other Therapeutic Products), Simvastatine (con.), Simvastatine (ngx) (simvastatin) Film-coated Tablet, Simvastatine (simvastatin) (simvastatin), Simvastatine (simvastatin) (tablets), Simvastatine (simvastatin) (tablets) (simvastatin), Simvastatine (simvastatine), Simvastatine (tablets), Simvastatine (zocor), Simvastatine 20 Ranbaxy Filmohulde Tabletten 20mg, Simvastatine 40 Mg, Simvastatine A, Simvastatine A (simvastatin), Simvastatine Asol, Simvastatine Aurobindo, Simvastatine Bexal, Simvastatine Eg, Simvastatine Nos, Simvastatine Pch Coated, Simvastatine Pch Tablet Filmomhuld 20 G (simvastatin), Simvastatine Pfizer, Simvastatine Ranbaxy 5mg (simvastatin) Tablet, 5 Mg, Simvastatine Teva, Simvastatinj, Simvastatinum, Simvastation, Simvastation 40 Mg, Simvastatn, Simvastatn (simvastatin) (simvastatin), Simvastatn 80 Mg, Simvastatni, Simvastatni (simvastatin), Simvastatom, Simvastatsn, Simvasterol, Simvasterol (simvastatin), Simvastidine, Simvastiin, Simvastin, Simvastin (simvastatin), Simvastin (con.), Simvastin (simavstatin), Simvastin (simvastatin), Simvastin (simvastatin) (unknown), Simvastin (simvastin) (simvastin), Simvastin -simvastatin- 10mg Lupin, Simvastin 40 Mg 40 Mg Dr. Reddys, Simvastin, Generic Zocar, Simvastin-mepha, Simvastin-mephra, Simvastine, Simvastitin, Simvaststin, Simvastten, Simvasttin, Simvasttin (simvastatin), Simvasttin 20mg, Simvasttin(simvastatin), Simvasvatin, Simvatad, Simvatatin, Simvatatin (con.), Simvatatin (simvastatin), Simvatatin (simvastatin) (simvastatin), Simvatatine, Simvatin (con.), Simvax, Simvaxtatin, Simviastatin, Simvist, Simvistatin, Simvistatin 20 Mg Generic, Simvistatin 20mg, Simvistatin Asa, Simvor, Simvor (simvastatin), Simvostatin, Simvstatin, Simvuastatin, Simvustatin, Simzastatin, Sinvacor, Sinvacor (20 Mg) (simvastatin), Sinvacor (simvastatin Ratiopharm), Sinvacor (simvastatin), Sinvacor (simvastatin) (1 Dosafe Forms, Tablets) (simvastatin), Sinvacor (simvastatin) (tablets) (simvastatin), Sinvacor (simvastatin, ), Sinvacor (simvastatln), Sinvalip (simvastatin) (simvastatin), Sinvascor, Sinvastacor, Sinvastacor (ngx), Sinvastacor (ngx) (simvastatin) Unknown, 40mg, Sinvastacor (ngx) (simvastatin) Unknown, 40mg, Sinvastacor (simvastatin), Sinvastanin, Sinvastatin, Sinvastatin (simvastatin), Sinvastatin (ta), Sinvastatin [simvastatin] (simvastatin), Sinvastatina, Sinvastatina (simvastatin), Sinvastatina Mepha, Sinvastatina Mepha (simvastatin), Sinvastatina Winthrop, Sinvastatine, Sinvastatine (simvastatin), Sinvastatine (simvastatine), Sinvastatine (smivastatin), Sinvastin, Sinvostetin, Siomvastatin, Sivastatin, Sivastatin (simvastatin), Sivastin, Sivastin (simvastatin Ratiopharm), Sivastin (simvastatin) (simvastatin), Sivastin (simvastatin) 40 Mg, Sivastin - (simvastatin) - Tablet - 40 Mg, Sivvastattin, Smivastatin, Smivastatin (simvastatin), Smvastatin, Smvastatin (simvastatin), Snvacor (simvastatin), Somvastatine (simvastatin), Statin (simvastatin), Stimvastatin, Stimvistatin, Stmvastattn (stmvastattn), Study Zocor-simvastatin, Sumvastatin, Symcora (simvastatin) (tablets), Symcora (simvastatin) (tablets), Symvastatin, Symvastatin -simvastatin- 20 Mg Rnb, Symvastin, Synovastatin, Synvastatin, Synvinolin (simvastatin), Synvostatin, Tab Placebo (unspecified), Tab Simvastatin, Tab Simvastatin 20 Mg, Vabadin (simvastatin), Vasilip, Vasilip (simvastatin), Vasilip (simvastatin) Tablet, Vaslip, Vaslip (simvastatin), Z0ocor (simvastatin), Zecor, Zicor, Zocar, Zocar (zocor Cardio Ass) Tablets, Zoclor, Zocoe (simvastatin), Zocol, Zocor, Zocor (simvastatin Ratiopharm), Zocor (zocor Cardio Ass), Zocor ^merck Frosst^, Zocor (simvastatin Ratiopharm), Zocor 10 Mg Merc, Zocor (40 Milligram), Zocor (all Other Therapeutic Products), Zocor (con. ), Zocor (con.), Zocor (generic), Zocor (simastatin), Zocor (simvastani), Zocor (simvastatin Ratiopharm), Zocor (simvastatin (simvastatin), Zocor (simvastatin Ratiopharm), Zocor (simvastatin Ratiophram), Zocor (simvastatin Rtiopharm), Zocor (simvastatin Sodium), Zocor (simvastatin) (10 Milligram), Zocor (simvastatin) (20 Milligram), Zocor (simvastatin) (40 Milligram), Zocor (simvastatin) (40 Milligram) (simvastatin), Zocor (simvastatin) (40 Milligram, Tablets), Zocor (simvastatin) (75 Milligram), Zocor (simvastatin) (80 Milligram, Tablets), Zocor (simvastatin) (simvastatin), Zocor (simvastatin) (tablets) (simvastatin), Zocor (simvastatin) (tablets) (simvastatin0, Zocor (simvastatin) (unknown), Zocor (simvastatin) 02/19/2011 To Unk, Zocor (simvastatin) Tablet, 40 Mg, Zocor (simvastatin) Unknown Unk To Unk, Zocor (simvastatin)(10 Milligram, Tablet) (simvastatin), Zocor (simvastatin)(unknown), Zocor (simvastatin), Unspecified, Unknown, Zocor (simvastatin)s, Zocor (simvastatn), Zocor (simvasttin Ratiopharm), Zocor (sinvastatin), Zocor (sivastatin), Zocor (smvastatin), Zocor (ta), Zocor (tablets) Simvastatin, Zocor (tablets) Simvastatin), Zocor (taf), Zocor (zocor Cardio Ass), Zocor -simvasatin-, Zocor 10 Mg Merc, Zocor 200mg, Zocor 40 (simvastatin), Zocor 40 Mg Daily Po, Zocor 40mg, Tabletten, Zocor 5mg, Tabletten, Zocor 80 Mgm, Zocor 9simvastatin), Zocor Cardiem Ass (acetylsalicylic Acid, Simvastatin), Zocor Cardio Ass (acetylsalicylic Acid), Zocor Cardio Ass (simvastatin), Zocor Eq, Zocor Forte (simvastatin) Unknown, Zocor Xl, Zocor ^dieckamnn^ (simvastatin), Zocor ^dieckmann^ (simvastatn), Zocor ^dieckman^, Zocor ^diekmann^, Zocor ^merck Forsst^ (simvastatin), Zocor ^merck Frosst^, Zocor ^merck Frosst^ (simvastatin), Zocor ^merck^ (simvatatin), Zocor ^merc^, Zocor ^msp^, Zocor ^ms^, Zocor ^neopharmed^ (simvastatin), Zocor(simvastatin Ratiopharm), Zocor(simvastatin) (80 Milligram), Zocor(simvastatin) Tablet, Iu, Zocor(smvastatin), Zocor(zocor Cardio Ass), Zocor-generic, Zocord, Zocord (simvastatin Ratiopharm), Zocord (simvastatin Ratiopharm), Zocord (simvastatin Ratiopharm), Zocord (simvastatin), Zocord (simvastatin) Tablets, Zocord. Mfr: Not Specified Dates: , Unknown, Zocoro (simvastatin), Zocort, Zocort (simvastatin), Zocort Hc, Zocr, Zoeor, Zokor, Zoocor ^merck^, Zoor, Zorcor (zocor Cardio Ass), Zorcor 20mg Merck+co, Zovor (simvastatin), Zoxor, Zoxor (simvastatin), Zpcpr (simvastatin)
