# Supplementary material for: A Survey of the FDA's AERS Database Regarding Muscle and Tendon Adverse Events Linked to the Statin Drug Class
Source: PLoS One. 2012 Aug 22;7(8):e42866. doi: 10.1371/journal.pone.0042866 (PMC3425581; doi:10.1371/journal.pone.0042866)
Supplement: File S2 — AERS – Adverse Events Reporting System. This file S2 delineates AERS search terms used for each adverse event category. (DOC) [file pone.0042866.s002.doc]

**Supplement** **S2**

#### Adverse Event Categories - AERS Search Terms Used

“Myopathy”

Myopathy

#### “Myalgia”

Myalgia

“Myositis”

Myositis

“Rhabdomyolysis”

Rhabdomyolysis

“Joints and Tendons”

arthralgia, arthropathy, joint ankylosis, joint contracture, joint destruction, joint hyperextension, joint injury, joint instability, joint ligament rupture, joint lock, joint range of motion decreased, joint sprain, joint stiffness, joint swelling, ligament disorder, ligament laxity, ligament pain, ligament repair, ligament rupture, ligament sprain, ligamentitis, purulent synovitis, synovitis, tendon injury, tendon necrosis, tendon operation, tendon pain, tendon repair, tendon rupture, tendonitis, tenosynovitis , tenosynovitis stenosans, trigger finger

“Muscle Atrophy and Injury”

amyotrophy, grip strength, grip strength decreased, muscle atrophy, muscle fibrosis, muscle haemorrhage, muscle injury, muscle necrosis, muscle rupture, neuromuscular toxicity, papillary muscle haemorrhage, papillary muscle infarction, papillary muscle rupture, progressive muscular atrophy, sarcopenia, spinal muscular atrophy

“Muscle Coordination and Weakness”

abasia, akinaesthesia, akinesia, amimia, asthenia, ataxia, athetosis, ballismus, bradykinesia, cachexia, chorea, choreoathetosis, clonus, cogwheel rigidity, coordination abnormal, diplegia, dyskinesia, dysstasia, dystonia, facial paresis, fall, fibromyalgia, gait apraxia, gait deviation, gait disturbance, gait hemiplegic, gait spastic, gross motor delay, hemiparesis, hemiplegia, hemiplegia transient, hyperkinesia, hyperreflexia, hypertonia, hypokinesia, hyporeflexia, hypotonia, immobile, immobilisation prolonged, latent tetany, limb immobilisation, locked-in syndrome, mobility decreased, monoparesis, monoplegia, movement disorder, muscle contracture, muscle cramp, muscle disorder, muscle fatigue, muscle operation, muscle rigidity, muscle spasms, muscle spasticity, muscle strain, muscle swelling, muscle tightness, muscle twitching, muscular dystrophy, muscular weakness, musculoskeletal disorder, musculoskeletal stiffness, myoclonus, myotonia, paralysis, paralysis flaccid, paralytic gait, paraparesis, paraplegia, paresis, parkinsonian gait, peripheral paralysis, quadriparesis, quadriplegia, respiratory muscle weakness, tetany
