# Supplementary material for: A Survey of the FDA's AERS Database Regarding Muscle and Tendon Adverse Events Linked to the Statin Drug Class
Source: PLoS One. 2012 Aug 22;7(8):e42866. doi: 10.1371/journal.pone.0042866 (PMC3425581; doi:10.1371/journal.pone.0042866)
Supplement: Table S1 — AEs – Adverse Events. For each muscle adverse event category this table lists the number of major clinical outcomes (e.g., death, disability, hospitalization) associated with that outcome, for each statin agent. (DOC) [file pone.0042866.s003.doc]

**Table S1**

Outcome Measures

**Myalgia**

Death

| **Drug Name** | **AEs**  **Primary / All** | **Ranked Risk** |
| --- | --- | --- |
| Fluvastatin | 6 / 9 | 100 |
| Rosuvastatin | 7 / 17 | 11 |
| Atorvastatin | 17 / 39 | 9 |
| Simvastatin | 21 / 57 | 9 |
| Lovastatin | 1 / 2 | 2 |
| Pravastatin | 1 / 8 | 2 |

Disability

| **Drug Name** | **AEs**  **Primary / All** | **Ranked Risk** |
| --- | --- | --- |
| Rosuvastatin | 92 / 135 | 100 |
| Atorvastatin | 192 / 304 | 70 |
| Fluvastatin | 5 / 15 | 60 |
| Simvastatin | 177 / 261 | 56 |
| Pravastatin | 15 / 64 | 21 |
| Lovastatin | 7 /19 | 10 |

Hospitalization – Initial or Prolonged

| **Drug Name** | **AEs**  **Primary / All** | **Ranked Risk** |
| --- | --- | --- |
| Fluvastatin | 29 / 51 | 100 |
| Rosuvastatin | 150 / 211 | 47 |
| Atorvastatin | 237 / 465 | 25 |
| Simvastatin | 255 / 502 | 23 |
| Pravastatin | 24 / 77 | 10 |
| Lovastatin | 11 / 37 | 5 |

Life-Threatening

| **Drug Name** | **AEs**  **Primary / All** | **Ranked Risk** |
| --- | --- | --- |
| Fluvastatin | 6 / 6 | 100 |
| Rosuvastatin | 20 / 32 | 30 |
| Simvastatin | 56 / 89 | 25 |
| Atorvastatin | 46 / 74 | 24 |
| Pravastatin | 12 / 21 | 23 |
| Lovastatin | 5 / 9 | 10 |

Required Intervention to Prevent Permanent Impairment/Damage

| **Drug Name** | **AEs**  **Primary / All** | **Ranked Risk** |
| --- | --- | --- |
| Rosuvastatin | 61 / 68 | 100 |
| Fluvastatin | 2 / 5 | 36 |
| Simvastatin | 64 / 88 | 30 |
| Atorvastatin | 50 / 69 | 28 |
| Pravastatin | 12 / 16 | 25 |
| Lovastatin | 8 / 11 | 18 |

**Myopathy**

Death

| **Drug Name** | **AEs**  **Primary / All** | **Ranked Risk** |
| --- | --- | --- |
| Simvastatin | 19 / 25 | 100 |
| Atorvastatin | 4 / 12 | 24 |
| Lovastatin | 1 / 6 | 24 |
| Fluvastatin | 0 / 1 | 0 |
| Rosuvastatin | 0 / 0 | 0 |
| Pravastatin | 0 / 2 | 0 |

Disability

| **Drug Name** | **AEs**  **Primary / All** | **Ranked Risk** |
| --- | --- | --- |
| Atorvastatin | 41 / 56 | 100 |
| Rosuvastatin | 11 / 13 | 80 |
| Fluvastatin | 1 / 3 | 79 |
| Simvastatin | 37 / 52 | 78 |
| Lovastatin | 0 / 0 | 0 |
| Pravastatin | 0 / 6 | 0 |

Hospitalization – Initial or Prolonged

| **Drug Name** | **AEs**  **Primary / All** | **Ranked Risk** |
| --- | --- | --- |
| Rosuvastatin | 34 / 42 | 100 |
| Fluvastatin | 3 / 6 | 97 |
| Simvastatin | 100 / 151 | 85 |
| Atorvastatin | 45 / 85 | 45 |
| Lovastatin | 7 / 11 | 28 |
| Pravastatin | 1 / 18 | 4 |

Life-Threatening

| **Drug Name** | **AEs**  **Primary / All** | **Ranked Risk** | |  |
| --- | --- | --- | --- | --- |
| Simvastatin | 24 / 29 | 100 | |  |
| Rosuvastatin | 6 / 6 | 86 | |  |
| Atorvastatin | 12 / 22 | 58 | |  |
| Lovastatin | 1 / 1 | 19 | |  |
| Pravastatin | 0 / 1 | | 0 | |
| Fluvastatin | 0 / 0 | | 0 | |

Required Intervention to Prevent Permanent Impairment/Damage

| **Drug Name** | **AEs**  **Primary / All** | **Ranked Risk** |
| --- | --- | --- |
| Rosuvastatin | 6 / 6 | 100 |
| Lovastatin | 2 / 3 | 45 |
| Atorvastatin | 6 / 9 | 34 |
| Simvastatin | 5 / 7 | 24 |
| Pravastatin | 0 / 1 | 0 |
| Fluvastatin | 0 / 0 | 0 |

**Myositis**

Death

| **Drug Name** | **AEs**  **Primary / All** | **Ranked Risk** |
| --- | --- | --- |
| Simvastatin | 7 / 14 | 100 |
| Pravastatin | 1 / 3 | 63 |
| Rosuvastatin | 1 / 1 | 49 |
| Atorvastatin | 2 / 5 | 33 |
| Lovastatin | 0 / 0 | 0 |
| Fluvastatin | 0 / 1 | 0 |

Disability

| **Drug Name** | **AEs**  **Primary / All** | **Ranked Risk** |
| --- | --- | --- |
| Fluvastatin | 1 / 1 | 100 |
| Simvastatin | 35 / 42 | 93 |
| Rosuvastatin | 8 / 9 | 73 |
| Atorvastatin | 15 / 22 | 46 |
| Pravastatin | 1 / 2 | 12 |
| Lovastatin | 0 / 0 | 0 |

Hospitalization – Initial or Prolonged

| **Drug Name** | **AEs**  **Primary / All** | **Ranked Risk** |
| --- | --- | --- |
| Simvastatin | 114 / 153 | 100 |
| Rosuvastatin | 22 / 24 | 67 |
| Fluvastatin | 2 / 5 | 66 |
| Atorvastatin | 31 / 58 | 32 |
| Pravastatin | 4 / 11 | 15 |
| Lovastatin | 1 / 2 | 4 |

Life-Threatening

| **Drug Name** | **AEs**  **Primary / All** | **Ranked Risk** |
| --- | --- | --- |
| Simvastatin | 29 / 30 | 100 |
| Rosuvastatin | 3 / 3 | 36 |
| Fluvastatin | 0 / 0 | 0 |
| Lovastatin | 0 / 0 | 0 |
| Pravastatin | 0 / 2 | 0 |
| Atorvastatin | 0 / 4 | 0 |

Required Intervention to Prevent Permanent Impairment/Damage

| **Drug Name** | **AEs**  **Primary / All** | **Ranked Risk** |
| --- | --- | --- |
| Rosuvastatin | 3 / 3 | 100 |
| Simvastatin | 10 / 10 | 97 |
| Atorvastatin | 3 / 4 | 34 |
| Lovastatin | 0 / 0 | 0 |
| Pravastatin | 0 / 0 | 0 |
| Fluvastatin | 0 / 0 | 0 |

**Rhabdomyolysis**

Death

| **Drug Name** | **AEs**  **Primary / All** | **Ranked Risk** |
| --- | --- | --- |
| Fluvastatin | 5 / 13 | 100 |
| Simvastatin | 152 / 215 | 80 |
| Atorvastatin | 91 / 134 | 56 |
| Rosuvastatin | 23 / 31 | 42 |
| Pravastatin | 10 / 29 | 23 |
| Lovastatin | 2 / 11 | 5 |

Disability

| **Drug Name** | **AEs**  **Primary / All** | **Ranked Risk** |
| --- | --- | --- |
| Simvastatin | 133 / 167 | 100 |
| Fluvastatin | 3 / 3 | 85 |
| Rosuvastatin | 26 / 28 | 67 |
| Atorvastatin | 47 / 84 | 41 |
| Lovastatin | 5 / 5 | 17 |
| Pravastatin | 5 / 15 | 17 |

Hospitalization – Initial or Prolonged

| **Drug Name** | **AEs**  **Primary / All** | **Ranked Risk** |
| --- | --- | --- |
| Fluvastatin | 31 / 67 | 100 |
| Rosuvastatin | 296 / 354 | 87 |
| Simvastatin | 1,000 / 1,371 | 85 |
| Atorvastatin | 316 / 542 | 31 |
| Pravastatin | 50 / 125 | 19 |
| Lovastatin | 41 / 73 | 16 |

Life-Threatening

| **Drug Name** | **AEs**  **Primary / All** | **Ranked Risk** |
| --- | --- | --- |
| Simvastatin | 288 / 383 | 100 |
| Fluvastatin | 6 / 23 | 79 |
| Rosuvastatin | 65 / 75 | 78 |
| Atorvastatin | 76 / 130 | 31 |
| Lovastatin | 11 / 13 | 18 |
| Pravastatin | 7 / 25 | 11 |

Required Intervention to Prevent Permanent Impairment/Damage

| **Drug Name** | **AEs**  **Primary / All** | **Ranked Risk** |
| --- | --- | --- |
| Rosuvastatin | 32 / 36 | 100 |
| Simvastatin | 76 / 94 | 69 |
| Atorvastatin | 20 / 24 | 21 |
| Lovastatin | 5 / 7 | 21 |
| Pravastatin | 5 / 6 | 20 |
| Fluvastatin | 0 / 1 | 0 |

### Combined Outcomes for Myalgia, Myopathy, Myositis, Rhabdomyolysis

Death

| **Drug Name** | **AEs**  **Primary / All** | **Ranked Risk** |
| --- | --- | --- |
| Fluvastatin | 9 / 19 | 100 |
| Simvastatin | 169 / 265 | 50 |
| Atorvastatin | 107 / 180 | 37 |
| Rosuvastatin | 28 / 46 | 28 |
| Pravastatin | 11 / 38 | 14 |
| Lovastatin | 3 / 15 | 4 |

Disability

| **Drug Name** | **AEs**  **Primary / All** | **Ranked Risk** |
| --- | --- | --- |
| Rosuvastatin | 125 / 173 | 100 |
| Simvastatin | 347 / 477 | 81 |
| Fluvastatin | 9 / 19 | 79 |
| Atorvastatin | 255 / 404 | 69 |
| Pravastatin | 19 / 79 | 19 |
| Lovastatin | 11 / 23 | 12 |

Hospitalization – Initial or Prolonged

| **Drug Name** | **AEs**  **Primary / All** | **Ranked Risk** |
| --- | --- | --- |
| Fluvastatin | 52 / 109 | 100 |
| Rosuvastatin | 457 / 580 | 80 |
| Simvastatin | 1,297 / 1,921 | 66 |
| Atorvastatin | 563 / 1,026 | 33 |
| Pravastatin | 75 / 212 | 17 |
| Lovastatin | 54 / 113 | 13 |

Life-Threatening

| **Drug Name** | **AEs**  **Primary / All** | **Ranked Risk** |
| --- | --- | --- |
| Fluvastatin | 10 / 27 | 100 |
| Simvastatin | 350 / 479 | 93 |
| Rosuvastatin | 84 / 104 | 77 |
| Atorvastatin | 114 / 196 | 35 |
| Pravastatin | 19 / 47 | 22 |
| Lovastatin | 16 / 22 | 20 |

Required Intervention to Prevent Permanent Impairment/Damage

| **Drug Name** | **AEs**  **Primary / All** | **Ranked Risk** |
| --- | --- | --- |
| Rosuvastatin | 99 / 110 | 100 |
| Simvastatin | 145 / 189 | 42 |
| Atorvastatin | 75 / 101 | 26 |
| Fluvastatin | 2 / 6 | 22 |
| Pravastatin | 16 / 22 | 21 |
| Lovastatin | 14 / 20 | 19 |

# Combined Adverse Outcomes (Death, Disability, Hospitalization, Life-Threatening, and Required Intervention) for Combined Muscle-Tendon Outcomes (Myalgia, Myositis, Myositis, Rhabdomyolysis).

| **Drug Name** | **AEs**  **Primary / All** | **Ranked Risk** |
| --- | --- | --- |
| Fluvastatin | 82 / 179 | 100 |
| Rosuvastatin | 793 / 1013 | 88 |
| Simvastatin | 2308 / 3331 | 75 |
| Atorvastatin | 1114 / 1907 | 42 |
| Pravastatin | 140 / 408 | 20 |
| Lovastatin | 98 / 193 | 15 |
