# Supplementary material for: A Survey of the FDA's AERS Database Regarding Muscle and Tendon Adverse Events Linked to the Statin Drug Class
Source: PLoS One. 2012 Aug 22;7(8):e42866. doi: 10.1371/journal.pone.0042866 (PMC3425581; doi:10.1371/journal.pone.0042866)
Supplement: Table S2 — This shows the percent contribution to statin adverse event reports by different groups (e.g., physician, pharmacist, consumer), stratified by adverse event category and statin drug. (DOC) [file pone.0042866.s004.doc]

**Table S2**

Reporter Identification - Percent Contributions to Case Report Totals

All Adverse Events

| Occupation | Rosuvastatin | Simvastatin | Atorvastatin | Lovastatin | Fluvastatin | Pravastatin |
| --- | --- | --- | --- | --- | --- | --- |
| Physician | 24.1% | 16.6% | 18.9% | 22.2% | 42.4% | 15.7% |
| Pharmacist | 3.7% | 12.3% | 7.1% | 17.4% | 11.1% | 7.9% |
| Other Health Professional | 7.5% | 36.9% | 12.9% | 12.5% | 14.4% | 21.2% |
| Consumer | 39.6% | 10.8% | 44.4% | 23.9% | 14.9% | 43.6% |
| Lawyer | 0.2% | 0.4% | 0.3% | 0.3% | 0.2% | 0.2% |
| No Data | 25.0% | 23.0% | 16.4% | 23.6% | 17.0% | 11.4% |

Myalgia

| Occupation | Rosuvastatin | Simvastatin | Atorvastatin | Lovastatin | Fluvastatin | Pravastatin |
| --- | --- | --- | --- | --- | --- | --- |
| Physician | 19.0% | 17.2% | 14.7% | 18.5% | 25.0% | 11.0% |
| Pharmacist | 5.2% | 16.8% | 6.0% | 21.5% | 18.2% | 14.0% |
| Other Health Professional | 6.1% | 27.0% | 11.7% | 15.4% | 15.9% | 16.9% |
| Consumer | 39.9% | 15.2% | 50.0% | 18.5% | 15.9% | 48.7% |
| Lawyer | 0.3% | 0.4% | 0.2% | 0.0% | 0.0% | 0.0% |
| No Data | 29.5% | 23.4% | 17.4% | 26.2% | 25.0% | 9.3% |

Myopathy

| Occupation | Rosuvastatin | Simvastatin | Atorvastatin | Lovastatin | Fluvastatin | Pravastatin |
| --- | --- | --- | --- | --- | --- | --- |
| Physician | 54.2% | 21.4% | 40.4% | 46.7% | 12.5% | 8.3% |
| Pharmacist | 10.2% | 10.4% | 3.7% | 6.7% | 12.5% | 8.3% |
| Other Health Professional | 3.4% | 37.0% | 15.4% | 13.3% | 37.5% | 50.0% |
| Consumer | 11.9% | 7.1% | 16.9% | 20.0% | 12.5% | 16.7% |
| Lawyer | 1.7% | 0.0% | 2.9% | 0.0% | 0.0% | 0.0% |
| No Data | 18.6% | 24.0% | 20.6% | 13.3% | 25.0% | 16.7% |

Myositis

| Occupation | Rosuvastatin | Simvastatin | Atorvastatin | Lovastatin | Fluvastatin | Pravastatin |
| --- | --- | --- | --- | --- | --- | --- |
| Physician | 67.5% | 9.5% | 54.7% | 33.3% | 20.0% | 36.4% |
| Pharmacist | 12.5% | 16.4% | 4.0% | 0.0% | 20.0% | 27.3% |
| Other Health Professional | 15.0% | 45.5% | 12.0% | 33.3% | 20.0% | 36.4% |
| Consumer | 2.5% | 3.2% | 16.0% | 33.3% | 0.0% | 0.0% |
| Lawyer | 0.0% | 0.0% | 0.0% | 0.0% | 0.0% | 0.0% |
| No Data | 2.5% | 25.4% | 13.3% | 0.0% | 40.0% | 0.0% |

Rhabdomyolysis

| Occupation | Rosuvastatin | Simvastatin | Atorvastatin | Lovastatin | Fluvastatin | Pravastatin |
| --- | --- | --- | --- | --- | --- | --- |
| Physician | 59.9% | 21.6% | 44.5% | 31.6% | 32.6% | 26.6% |
| Pharmacist | 14.7% | 25.7% | 11.6% | 40.4% | 9.3% | 20.3% |
| Other Health Professional | 11.4% | 31.9% | 24.4% | 10.5% | 27.9% | 34.4% |
| Consumer | 5.0% | 2.7% | 7.4% | 3.5% | 4.7% | 12.5% |
| Lawyer | 1.2% | 0.4% | 0.4% | 0.0% | 0.0% | 0.0% |
| No Data | 7.8% | 17.7% | 11.8% | 14.0% | 25.6% | 6.3% |

Joints and Tendons

| Occupation | Rosuvastatin | Simvastatin | Atorvastatin | Lovastatin | Fluvastatin | Pravastatin |
| --- | --- | --- | --- | --- | --- | --- |
| Physician | 16.6% | 8.8% | 13.8% | 9.1% | 22.9% | 16.5% |
| Pharmacist | 3.2% | 6.2% | 4.8% | 9.1% | 8.6% | 6.2% |
| Other Health Professional | 7.6% | 35.3% | 8.1% | 6.1% | 5.7% | 22.7% |
| Consumer | 42.1% | 21.5% | 54.8% | 60.6% | 25.7% | 35.1% |
| Lawyer | 0.2% | 0.7% | 0.2% | 0.0% | 0.0% | 1.0% |
| No Data | 30.2% | 27.5% | 18.3% | 15.2% | 37.1% | 18.6% |

Muscle Atrophy and Injury

| Occupation | Rosuvastatin | Simvastatin | Atorvastatin | Lovastatin | Fluvastatin | Pravastatin |
| --- | --- | --- | --- | --- | --- | --- |
| Physician | 29.9% | 18.4% | 22.5% | 5.3% | 37.5% | 29.3% |
| Pharmacist | 2.8% | 2.2% | 5.3% | 0.0% | 0.0% | 0.0% |
| Other Health Professional | 2.8% | 35.3% | 5.6% | 2.6% | 6.3% | 22.0% |
| Consumer | 34.6% | 18.4% | 46.5% | 5.3% | 18.8% | 24.4% |
| Lawyer | 1.9% | 1.5% | 0.6% | 0.0% | 0.0% | 0.0% |
| No Data | 28.0% | 24.3% | 19.6% | 86.8% | 37.5% | 24.4% |

Muscle Coordination and Weakness

| Occupation | Rosuvastatin | Simvastatin | Atorvastatin | Lovastatin | Fluvastatin | Pravastatin |
| --- | --- | --- | --- | --- | --- | --- |
| Physician | 14.5% | 11.2% | 12.4% | 14.4% | 31.9% | 7.2% |
| Pharmacist | 2.6% | 11.8% | 5.1% | 9.3% | 5.9% | 5.5% |
| Other Health Professional | 6.3% | 32.1% | 8.8% | 13.6% | 19.3% | 17.7% |
| Consumer | 46.5% | 19.7% | 50.7% | 39.0% | 24.4% | 50.6% |
| Lawyer | 0.3% | 0.5% | 0.6% | 0.0% | 0.8% | 0.0% |
| No Data | 29.8% | 24.7% | 22.4% | 23.7% | 17.6% | 19.0% |
