# Supplementary material for: A Survey of the FDA's AERS Database Regarding Muscle and Tendon Adverse Events Linked to the Statin Drug Class
Source: PLoS One. 2012 Aug 22;7(8):e42866. doi: 10.1371/journal.pone.0042866 (PMC3425581; doi:10.1371/journal.pone.0042866)
Supplement: Table S3 — *Based on similar LDL reduction [30] , [31] . Note: These are inexact equivalency approximations. (DOC) [file pone.0042866.s005.doc]

**Table S3**

| Approximate Relative Dose Equivalence of Statins* | | | | | | |
| --- | --- | --- | --- | --- | --- | --- |
| Rosuvastatin | Atorvastatin | Simvastatin | Lovastatin | Pravastatin | Fluvastatin |  |
| - | - | 5mg | 10mg | 10mg | 20mg |  |
| - | 5mg | 10mg | 20mg | 20mg | 40mg |  |
| 2.5-5mg | 10mg | 20mg | 40mg | 40mg | 80mg |  |
| 5-10mg | 20mg | 40mg | 80mg | 80mg | - |  |
| 10mg-20mg | 40mg | 80mg | - | - | - |  |
| 20-40mg | 80mg | - | - | - | - |  |
